# Structure-Activity Relationships of Pentacyclic Triterpenoids as Potent and Selective Inhibitors against Human Carboxylesterase 1

**DOI:** 10.3389/fphar.2017.00435

**Published:** 2017-06-30

**Authors:** Li-Wei Zou, Tong-Yi Dou, Ping Wang, Wei Lei, Zi-Miao Weng, Jie Hou, Dan-Dan Wang, Yi-Ming Fan, Wei-Dong Zhang, Guang-Bo Ge, Ling Yang

**Affiliations:** ^1^Institute of Interdisciplinary Integrative Medicine Research, Shanghai University of Traditional Chinese MedicineShanghai, China; ^2^Laboratory of Pharmaceutical Resource Discovery, Dalian Institute of Chemical Physics, Chinese Academy of SciencesDalian, China; ^3^School of Life Science and Medicine, Dalian University of TechnologyPanjin, China; ^4^Biotechnology Department, College of Basic Medical Sciences, Dalian Medical UniversityDalian, China

**Keywords:** human carboxylesterase 1 (hCE1), ursolic acid, oleanolic acid, structure-activity relationship (SAR), selective inhibitors

## Abstract

Human carboxylesterase 1 (hCE1), one of the most important serine hydrolases distributed in liver and adipocytes, plays key roles in endobiotic homeostasis and xenobiotic metabolism. This study aimed to find potent and selective inhibitors against hCE1 from phytochemicals and their derivatives. To this end, a series of natural triterpenoids were collected and their inhibitory effects against human carboxylesterases (hCEs) were assayed using D-Luciferin methyl ester (DME) and 6,8-dichloro-9,9-dimethyl-7-oxo-7,9-dihydroacridin-2-yl benzoate (DDAB) as specific optical substrate for hCE1, and hCE2, respectively. Following screening of a series of natural triterpenoids, oleanolic acid (OA), and ursolic acid (UA) were found with strong inhibitory effects on hCE1 and relative high selectivity over hCE2. In order to get the highly selective and potent inhibitors of hCE1, a series of OA and UA derivatives were synthesized from OA and UA by chemical modifications including oxidation, reduction, esterification, and amidation. The inhibitory effects of these derivatives on hCEs were assayed and the structure-activity relationships of tested triterpenoids as hCE1 inhibitors were carefully investigated. The results demonstrated that the carbonyl group at the C-28 site is essential for hCE1 inhibition, the modifications of OA or UA at this site including esters, amides and alcohols are unbeneficial for hCE1 inhibition. In contrast, the structural modifications on OA and UA at other sites, such as converting the C-3 hydroxy group to 3-O-β-carboxypropionyl (compounds **20** and **22**), led to a dramatically increase of the inhibitory effects against hCE1 and very high selectivity over hCE2. 3D-QSAR analysis of all tested triterpenoids including OA and UA derivatives provide new insights into the fine relationships linking between the inhibitory effects on hCE1 and the steric-electrostatic properties of triterpenoids. Furthermore, both inhibition kinetic analyses and docking simulations demonstrated that compound **22** was a potent competitive inhibitor against hCE1-mediated DME hydrolysis. All these findings are very helpful for medicinal chemists to design and develop highly selective and more potent hCE1 inhibitors for biomedical applications.

## Introduction

Mammalian carboxylesterases (CEs) are important members of the serine hydrolase superfamily (E.C. 3.1.1.1), which catalyze the hydrolysis of a wide variety of endogenous and xenobiotics ester compounds (Satoh and Hosokawa, 1998; Redinbo and Potter, 2005). In human, two primary carboxylesterases including human carboxylesterase 1 (hCE1) and human carboxylesterase 2 (hCE2), have been found and extensively studied in the past decade (Imai, 2006). These two isoforms share 47% amino acid sequence identity, but exhibit differential tissue distribution and distinct substrate and inhibitor specificities (Hosokawa, 2008). Generally, hCE1 is primarily expressed in the liver and adipocytes, and demonstrates substrate specificity for a large, bulky acyl group and a small alcohol group (Satoh et al., 2002; Imai et al., 2006). In contrast, hCE2 is highly expressed in the intestine and colon, and displays the opposite substrate preference for as mall acyl group and a large alcohol group (Xu et al., 2002; Sanghani et al., 2003; Kobayashi et al., 2012). The distribution and the catalytic property of hCE1 make this enzyme a key determinant for the bioactivation of numerous ester-containing drugs including oseltamivir (Shi et al., 2006), dabigatran etexilate (Hu et al., 2013), mycophenolate mofetil (Fujiyama et al., 2010), and trandolapril (Zhu et al., 2009), as well as for the metabolic inactivation and clearance of some esterified drugs, such as clopidogrel (Tang et al., 2006), methylphenidate (Sun et al., 2004), rufinamide (Williams et al., 2010), and oxybutynin (Sato et al., 2012).

As one of the most abundant esterases distributed in human liver and adipocytes, hCE1 participates in a wide range of physiological or pathological processes *via* hydrolysis of endogenous esters (such as cholesteryl esters and triacylglycerols) and thus plays key roles in cholesterol homeostasis and fatty acid metabolism (Crow et al., 2008; Li et al., 2016). Recent studies have revealed that the activities of hCE1 are markedly elevated in obese individuals and patients with type 2 diabetes, and the treatment of hCE1 inhibitors displayed multiple beneficial effects in both lipid and glucose homeostasis in genetic and diet-induced mouse models of obesity, insulin resistance and type 2 diabetes (Dominguez et al., 2014). Furthermore, hCE1 has been recognized as a therapeutic target for hypertriglyceridaemia, due to the key roles of this enzyme responsible for the enzymatic cleaving of triglyceride stores in hepatocytes (Gilham et al., 2003). The key roles of hCE1 in human diseases make the discovery of potent and selective inhibitors of hCE1 as drug candidates is of immense significance in both basic researches and clinical applications. However, the highly selective and potent inhibitors of hCE1 have been rarely reported. To data, only one hCE1 inhibitor termed GR148672X is in preclinical development for the treatment of hypertriglyceridaemia, but the selectivity and molecular interactions of this agent have not been disclosed (Gilham et al., 2003; Bachovchin and Cravatt, 2012). Thus, it is highly desirable to find more potent and selective hCE1 inhibitors for potential biomedical applications, including exploring the functions of hCE1 in biological systems and serving as therapeutic agents for the treatment of obese, type 2 diabetes and hypertriglyceridaemia.

In recent years, screening of the specific and potent inhibitors toward CEs from phytochemicals in medicinal plants or herbs has attracted increasing attentions (Liu et al., 2016; Wang et al., 2017), owing to most of phytochemicals display satisfying safety during long history of use for medical treatments (Li and Vederas, 2009; Ngo et al., 2013; Shen, 2015). To data, many phytochemicals including flavonoids (Li et al., 2015; Sun et al., 2016), tanshinones (Hatfield et al., 2013), and triterpenoids (Mai et al., 2015; Zou et al., 2016) have been reported with inhibitory effects against human carboxylesterases. However, most of these natural compounds demonstrated more potent inhibitory effects against hCE2 in contrast to hCE1 (Hatfield and Potter, 2011; Umehara et al., 2016; Xu et al., 2016; Wang et al., 2017). Thus, it is urgently necessary to find more potent and selective hCE1 inhibitors from phytochemicals. More recently, we have developed a highly specific bioluminescent probe substrate (termed DME) for hCE1 and a highly selective near-infrared fluorescent probe (termed DDAB) for hCE2, which have been successfully used for rapid screening and characterization of inhibitors against hCEs using cell or tissue preparations as enzyme sources (Jin et al., 2016; Wang et al., 2016). In the present study, DME and DDAB were used as the highly selective optical substrates for human CEs to rapidly screen hCE1 inhibitors from natural triterpenoid compounds. After preliminary screening, we found that two pentacyclic triterpenoids including oleanolic acid (OA) and ursolic acid (UA) displayed potent inhibitory effects against hCE1, with the IC_50_ values of 0.28 μM (for OA) and 0.24 μM (for UA), as well as relatively high selectivity over hCE2 (>19-folds). These findings promoted us to develop more potent and highly selective inhibitors against hCE1, using these two natural compounds as lead compounds. Hence, a series of OA and UA derivatives were semi-synthesized and assayed, while the structure-activity relationships (SAR) and 3D-QSAR analysis for all tested triterpenoids including OA and UA derivatives as hCE1 inhibitors were carefully studied. The obtained SAR was very helpful for the development of more potent and highly selective inhibitors against hCE1.

## Materials and methods

### Chemicals and reagents

Natural triterpenoids were purchased from Chengdu Pufei De Biotech Co., Ltd. (Chengdu, Sichuan, China). The purities of all tested compounds were determined by HPLC-UV, which were greater than 98%. Bis-*p*-nitrophenyl phosphate (BNPP) was purchased from TCI (Tokyo, Japan). Stock solutions of compound **1**–**27** were prepared in DMSO and stored at 4°C until use. Phosphate buffer (100 mM, pH 7.4 and pH 6.5) was prepared by using Millipore water and stored at 4°C until use. Human liver microsomes (HLMs) were obtained from Celsis (Shanghai, China). The specific probes DME (hCE1) and DDAB (hCE2) were synthesized in our lab as described previously (Jin et al., 2016; Wang et al., 2016). Millipore water (Millipore, Bedford, USA), HPLC grade acetonitrile, methanol, and formic acid (Tedia company, USA) were employed throughout the study. The Luciferin Detection Reagent (LDR) was obtained from Promega Corporation (USA). All ^1^H NMR (400 MHz) and ^13^C NMR (101 MHz) were recorded on a VARIAN INOVA-400 spectrometer with chemical shifts reported as ppm (in CDCl_3_, TMS as the internal standard). High resolution MS data were obtained with the LTQ Orbitrap mass spectrometer (Orbitrap Elite).

### Instrument and analytical methods

All fluorescence-based assays were performed on Synergy H^1^ Hybrid Multi-Mode Microplate Reader (BioTek, USA). The measurements for the hydrolytic metabolite of DME were set as follow, luminescence, gain = 135. The measurements for the hydrolytic metabolite of DDAB were set as follow, fluorescence detection with the excitation wavelength at 600 nm and the emission wavelength at 662 nm, gain = 100.

### Enzyme inhibition assays

The inhibitory effects toward CEs were assayed using DME and DDAB as the specific probe substrates for hCE1 and hCE2, respectively. In brief, for the inhibition assays with DDAB, the incubation mixture (200 μL total volume) consisted of PBS, enzyme sources (0.5 and 5 μg/mL, HLM), inhibitors (compounds **1**–**27** or BNPP) and DDAB (both 10 μM, final concentration). Control incubations without BNPP were also performed. After incubation, ice-cold acetonitrile (equal volume of incubation mixture, 200 μL) was added to terminate the reaction. The mixture was then centrifuged at 20,000 g for 20 min at 4°C. All these compounds dissolved in DMSO, and the final concentration of DMSO was <2% (V/V). Aliquots of the supernatant were then taken for further microplate reader analyses. For inhibition assays with probe DME, the incubation mixture (50 μL total volume) consisted of PBS, enzyme sources (10 μg/mL, HLM), inhibitors (compounds **1**–**27** or BNPP) and DME (3 μM, final concentration). First, each of the potential esterase inhibitors was pre-incubated with HLM at 37°C for 10 min with shaking in microplate reader. Then DME was added to start the reaction. After incubation at 37°C for 10 min in a shaking bath, LDR (equal volume of incubation mixture, 50 μL) was added to terminate the reaction, and luminescence measurements were conducted as described above. The residual enzymatic activity (%) was determined by the percent of D-fluorescein production in the presence of known selective esterase inhibitors to the control (in the absence of inhibitors).

### Inhibition kinetic analyses

The half maximal inhibitory concentration (IC_50_) of each compound was determined using various inhibitor concentrations under the same incubation conditions as mentioned above. The inhibition constant (*K*_i_) values of compound 22 against hCE1 in HLM were determined using varied concentrations of DME in the presence or absence of each inhibitor. To determine the inhibition kinetic types (competitive inhibition, non-competitive inhibition, or uncompetitive type) of tested compounds, multiple concentrations of DME and varied concentrations of inhibitor were utilized to determine the corresponding reaction rates. Dixon plot and Lineweaver-Burk plots were used to fit the data. The inhibition kinetic type was evaluated by determining the intersection point in the Dixon and Lineweaver-Burk plots. The second plots based on the slopes from Lineweaver-Burk plot vs. inhibitor concentrations were utilized to calculate each the inhibition constant (*K*_i_) value (Wang et al., 2015; Zhu et al., 2015, 2016).

### Statistical analysis

All values obtained from experiments were expressed as mean ± *SD*. The IC_50_ values which concentration of inhibitor that reduces enzyme activity by 50% and the *K*_i_ values were evaluated by nonlinear regression using GraphPad Prism 6.0 software (GraphPad Software, Inc., La Jolla, USA).

### Molecular docking

The protein structures of hCE1 were taken from the Protein Data Bank (PDB ID: 2DQY; Bencharit et al., 2003). The whole molecular docking process in this work was performed using Discovery Studio (BIOVIA Discovery Studio 2016, Dassault Systèmes, San Diego, USA). The “Prepare Protein” procedure was used to prepare the input protein structures for docking. Tasks including inserting missing atoms in incomplete residues, modeling missing loop regions, deleting alternate conformations (disorder), removing waters, standardizing atom names, and protonating titratable residues using predicted *pKs* were performed. Meanwhile, the “Prepare Ligand” procedure was used to prepare the input ligands for docking. Tasks including removing duplicates, enumerating isomers and tautomers, and generating 3D conformations were performed. The CHARMM 40.1 force field was used to represent the protein and ligand structures. Docking simulations were performed by a standard LibDock protocol, where protein features are referred to as hotspots. After a final energy-minimization step (allowing the ligand poses to be flexible), the top scoring ligand poses are saved. The rigid poses are placed into the active site of hCE1 and the hotspots are matched as triplets. For docking process, all ligands including DME and compound **22** are inputted, totally 431 conformers are generated, and finally 548 poses are successfully docked into the active site of hCE1. The protein-ligand complexes with the highest LibDock score were taken from the docking results and depicted in full text.

### 3D-QSAR models

Three 3D-QSAR models were built using the corresponding package of Drug Discovery Studio. Primarily, compound **1–27** were aligned by consensus on both steric and electrostatic fields, with relative weight of 50–50%. The aligned molecules were placed in a 3D grid space, with grid spacing of 1.5 Å. The extent of the grid was set to the bounding box of all the ligands plus 6.0 Å of extension. The CHARMm force field was used. The electrostatic potential and the van der Waals potential were treated as separate terms. A +1e point charge was used as the electrostatic potential probe. Distance-dependent dielectric constant was used to mimic the solvation effect. For the van der Waals potential, a carbon atom with a 1.73 Å radius was used as a probe. The energy grid potentials were filtered to remove highly correlated descriptors (maximum descriptor correlation was set as 0.9). Rather than the full potential, a soft-core potential suggested in CDOCKER (Wu et al., 2003) was used. The energy grid potentials were filtered to remove highly correlated descriptors. Partial least squares (PLS) models were then built using these remaining descriptors, and Log(IC_50_ for hCE1) was used as activity properties. Cross validation were performed by splitting the training data into five groups.

### General procedures for the synthesis of compounds 15–27

The NMR data of synthetic compounds are provided in the Supplementary Material.

#### 3-oxo-olean-12-en-28-oic acid (15)

To a solution of oleanoic acid **1** (456.7 mg, 1 mmol) in acetone (10 mL) was added Jones reagent (prepared from 107.9 mg of CrO_3_) at 0°C over a period of 30 min till the brown color persisted. The resulting solution was stirred for further 30 min. Progress of the reaction was monitored by TLC. After completion of the reaction, isopropanol (0.5 mL) was added. After evaporation of the solvent, the crude residue was diluted with dichloromethane (100 mL). The organic layer was washed with water (25 mL × 2), brine (25 mL) and dried over sodium sulfate. The solvent was concentrated under vacuum and the residual solid was purified by column chromatography on silica gel (petroleum ether/ethyl acetate = 20/1) to give the compound **15** (383.6 mg, 82%) as a white solid.

#### 3β-olean-12-ene-3,28-diol (16)

To a cooled (0°C) solution of LiAlH_4_ (75.9 mg, 2.0 mmol) in dry THF (8 mL) was added dropwise oleanoic acid **1** (235.4 mg, 0.5 mmol) in dry THF (5 mL) under Ar_2_ atmosphere. Suspension was stirred at 0°C for 1 h, allowed to rise to room temperature. Progress of the reaction was monitored by TLC. After completion of the reaction, the reaction was quenched with a solution of 1N NaOH (5 mL). Precipitate was filtered and washed with ethyl acetate. Organic phase was separated and aqueous layer was further extracted with ethyl acetate (25 mL × 2). The organic phase was washed with water (25 mL), brine (25 mL), and dried over sodium sulfate. After evaporation of the solvent, the crude residue was purified by column chromatography on silica gel (petroleum ether/ethyl acetate = 10/1) to give the compound **16** (123.9 mg, 56%) as a white solid.

#### 3β-hydroxy-olean-12-en-28-oic acidmethyl ester (17)

To a stirred solution of oleanoic acid **1** (228.4 mg, 0.5 mmol) in acetone (10 ml), anhydrous K_2_CO_3_ (69.1 mg, 0.5 mmol), and CH_3_I (46.7 μL, 0.75 mmol) were added at room temperature. The resulting solution was stirred for 12 h. Progress of the reaction was monitored by TLC. After completion of the reaction, the acetone was distilled off. The resulting mixture was diluted with water (15 mL) and extracted with dichloromethane (30 mL × 2). The combined organic layers were washed with brine (10 mL) and dried over sodium sulfate. After evaporation of the solvent, the crude residue was purified by column chromatography on silica gel (petroleum ether/ethyl acetate = 10/1) to give the compound **17** (203 mg, 86%) as a white solid.

#### 3β-hydroxy-olean-12-en-28-amide (18)

To a solution of compound **19** (260 mg, 0.52 mmol) in dichloromethane (10 mL), oxalylchloride (262 μL, 3.12 mmol) was added dropwise at room temperature. After stirring at room temperature for 2 h, the solvent was removed under reduced pressure and the residue was dissolved in toluene (10 mL). Then a conc. solution of ammonia (7 mL) was added at 4–8°C and the mixture was stirred for further 1 h. The mixture was extracted with dichloromethane (25 mL × 2). The combined organic layers were washed with water (10 mL), brine (10 mL), and dried over sodium sulfate. After evaporation of the solvent, the crude residue was purified by column chromatography on silica gel (petroleum ether/ethyl acetate = 5/1) to give a white solid. To a solution of the white solid (135 mg, 0.28 mmol) in methanol (6 mL) and tetrahydrofuran (2 mL), 1 M NaOH aq. (840 μL, 0.84 mmol) was added dropwise at room temperature. The resulting solution was stirred at 40°C for 5 h. Progress of the reaction was monitored by TLC. After completion of the reaction, the solvent was removed under reduced pressure. The resulting mixture was diluted with water (20 mL) and extracted with dichloromethane (30 mL × 2). The combined organic layers were washed with brine (15 mL) and dried over sodium sulfate. After evaporation of the solvent, the crude residue was purified by column chromatography on silica gel (dichloromethane/ methanol = 20/1) to give the compound **18** (100 mg, 42%) as a white solid.

#### 3β-O-acetyl-olean-12-en-28-oic acid (19)

To a stirred solution of oleanoic acid **1** (500 mg, 1.09 mmol) in pyridine (7.5 ml), acetic anhydride (5.0 mL) was added dropwise at 0°C. The resulting solution was stirred at room temperature for 24 h and then poured into ice water (75 mL), resulting in the compound **19** (514 mg, 92%) as white solid precipitate.

#### 3β-O-(β-carboxypropionyl)-olean-12-en-28-oic acid (20)

To a solution of oleanoic acid **1** (137 mg, 0.3 mmol) in dichloromethane (3 ml), succinic anhydride (150.1 mg, 1.5 mmol) and DMAP (73.3 mg, 0.6 mmol) were added at room temperature. The resulting solution was stirred at room temperature for 48 h. Progress of the reaction was monitored by TLC. After completion of the reaction, the mixture was acidified with 1 N HCl to pH~3 and extracted with ethyl acetate (30 mL × 3). The combined organic layers were washed with water (10 mL), brine (10 mL), and dried over sodium sulfate. After evaporation of the solvent, the crude residue was purified by column chromatography on silica gel (dichloromethane/ methanol = 20/1) to give the compound **20** (155 mg, 93%) as a white solid.

#### 3-oxo-urs-12-en-28-oic acid (21)

The preparation was performed as described above for compound **15** starting from ursoic acid **2** (182.8 mg, 0.4 mmol) to give compound **21** (134.2 mg, 74%) as a white solid.

#### 3β-O-(β-carboxypropionyl)-urs-12-en-28-oic acid (22)

The preparation was performed as described above for compound **20** starting from ursoic acid **2** (137 mg, 0.3 mmol) to give compound **22** (145 mg, 87%) as a white solid.

#### 3β-hydroxy-urs-12-en-28-oic acid methyl ester (23)

The preparation was performed as described above for compound **17** starting from ursoic acid **2** (456.7 mg, 1.0 mmol) to give compound **23** (450 mg, 95%) as a white solid.

#### 3-oxo-urs-12-en-28-oic acid methyl ester (24)

The preparation was performed as described above for compound **15** starting from compound **23** (94.2 mg, 0.2 mmol) to give compound **24** (92 mg, 98%) as a white solid.

#### 3,11-dioxo-urs-12-en-28-oic acid methyl ester (25)

To a solution of compound **24** (60 mg, 0.13 mmol) inacetic acid (2 ml) and acetic anhydride (2 ml), chromium trioxide (39.6 mg, 0.39 mmol) were added at room temperature. The resulting solution was stirred at rt for 3 h. Progress of the reaction was monitored by TLC. After completion of the reaction, the mixture was poured into a cooling water (20 mL). The mixture was adjusted to pH = 7~8 with 1 N NaOH, and then extracted with ethyl acetate (20 mL × 3). The combined organic layers were washed with water (10 mL), brine (10 mL), and dried over sodium sulfate. After evaporation of the solvent, the crude residue was purified by column chromatography on silica gel (dichloromethane/ methanol = 10/1) to give the compound **25** (31.3 mg, 50%) as a white solid.

#### 3β-O-(β-carboxypropionyl)-olean-12-en-30-oic acid (27)

Compound **26** was prepared from glycyrrhetinic acid according to the published literature (Zou et al., 2016). The preparation of compound **27** was performed as described above for compound **20** starting from compound **26** (68.5 mg, 0.15 mmol) to give a white solid (81.4 mg, 97%).

## Results and discussion

### Screening of hCE1 inhibitors from natural triterpenoids

**Figure 1:**
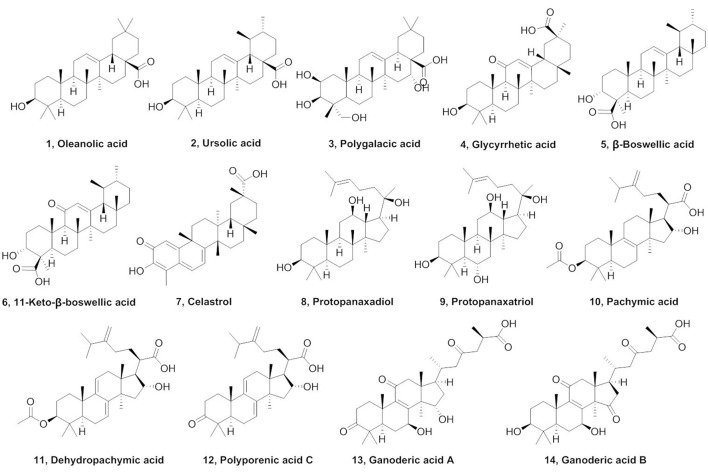
Chemical structures of tested natural triterpenoids.

Triterpenes, a class of widespread natural compounds containing six isoprene units, are an excellent reservoir of biologically active compounds (Sheng and Sun, 2011; Hill and Connolly, 2017). In this study, a series of natural triterpenoids were collected and their inhibitory effects against human carboxylesterases were assayed by using DME and DDAB as specific optical substrate for hCE1 and hCE2, respectively (Figure 1). The bioassay results are summarized in Table 1. It is evident from Table 1 that two natural triterpenoids including oleanolic acid (OA, 1) and ursolic acid (UA, 2) displayed potent inhibitory effects against hCE1 (IC_50_, 0.28 and 0.24 μM, respectively) and relative high selectivity over hCE2 (19.6- and 25.2-fold against hCE2, respectively). In contrast, other natural triterpenoids (3–14) displayed both poor selectivity and specificity toward hCE1.

**Table 1:** The IC_50_ values of natural triterpenoids toward hCE1 and hCE2.

**Compound**	**IC_50_ (hCE2)^a^μM**	**IC_50_ (hCE1)^a^μM**	Selectivity^*^
**1**	5.49	0.28	19.61
**2**	6.05	0.24	25.21
**3**	>100	64.74	>1.54
**4**	69.26	12.96	5.34
**5**	2.12	>400	<0.005
**6**	68.87	123.5	0.069
**7**	14.11	4.43	3.18
**8**	2.658	>500	<0.005
**9**	9.800	>500	<0.02
**10**	14.12	21.74	0.65
**11**	>100	27.61	>3.62
**12**	>100	58.6	>1.71
**13**	>100	>400	**–**
**14**	>100	>400	–

* *Selectivity is calculated from IC_50_ (hCE1)/IC_50_ (hCE2)*.

a *All data presented are averages of at least three separate experiments*.

### Chemistry

**Figure 2:**
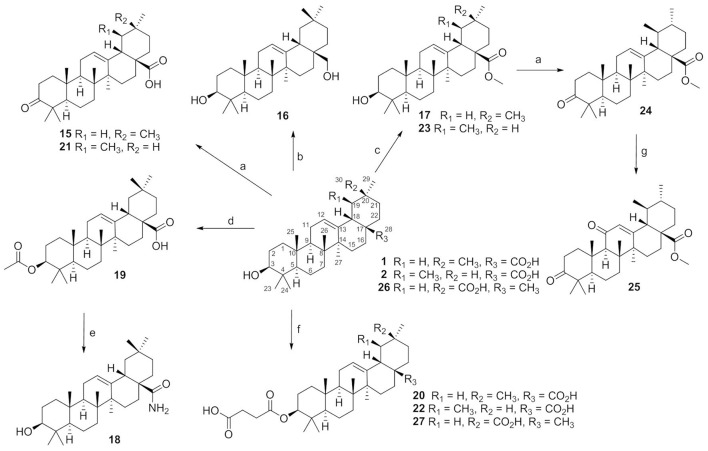
The synthesis routes for compounds 15–27. Reagents and conditions: (a) Jones reagent, acetone, 0°C, 1 h, 74–98%; (b) LiALH_4_, THF, rt, 24 h, 56%; (c) CH_3_I, K_2_CO_3_, acetone, rt, 12 h, 86–95%; (d) acetic anhydride, pyridine, rt, 12 h, 92%; (e) (COCl)_2_, CH_2_Cl_2_, rt, 2 h, then conc. ammonia, toluene, 4–8°C, NaOH, MeOH/THF, 40°C, 5 h, 42%; (f) succinic anhydride, DMAP, CH_2_Cl_2_, rt, 24 h, 87–97%; (g) CrO_3_, Ac_2_O, AcOH, rt, 3 h,50%.

Compounds **15**–**27** were semi-synthesized according to Figure 2. 3-Keto compounds **15** and **21** were obtained with the Jones' reagent in high yield from **1** (OA), and **2** (UA), respectively. The OA was reduced with lithium aluminum hydride to afford the 28-hydroxymethyl compound **16**. Reaction of the iodomethane with OA and UA furnished the target compounds **17** and **23**. OA was acetylated in C-3 with acetic anhydride in pyridine to obtain ester **19** with high yield (92%). Compound **19**, the acetate of OA, was then treated with oxalyl chloride without isolation, and further reacted with concentrated ammonia to afford amide compound which was hydrolyzed by NaOH to afford compound **18** in a yield of 42% over two steps. OA, UA and compound **26** were reacted with succinic anhydride in the presence of 4-dimethylaminopyridine to obtain the target product **20**, **22**, and **27**, respectively. Compound **24** was synthesized from **23** by the same method as compound **15**, with 98% yield. Compound **24** was oxidated with chromium trioxide to afford compound **25**.

### SAR study

Compounds **15**–**27** were assayed for their inhibitory effects against human CEs, including hCE1 and hCE2. The bioassay results were summarized in Table 2. Compound **15** exhibited relatively high selectivity toward hCE1 as compared with OA, suggesting that the introduction of carbonyl group at the C-3 site resulted in an increase of selectivity toward hCE1. Further modifications on the carbonyl group at the C-28 site of OA were conducted, and the alcohols (**16**), esters (**17**), and amides (**18**) derivatives were synthesized. As shown in Table 2, compounds **16**–**18** displayed both poor selectivity and specificity toward hCE1 compared with OA, suggesting that the carboxyl group at the C-28 site was very essential for hCE1 inhibition. Replacement of the C-3 hydroxyl group of OA with ethyl ester in compound **19** led to an increase of the inhibitory effects on hCE1 and a considerable selectivity toward hCE1 rather than hCE2, as compared with OA. Notably, replacement of the C-3 ethyl ester group with 3-O-β-carboxypropionyl in compound **20** led to a dramatically increase in the inhibitory effects against hCE1 (IC_50_, 17 nM) and the high selectivity over hCE2 (3296-fold against hCE2). These results suggested that the structural modifications on the C-3 hydroxyl group of OA were more feasible for the development of potent and highly selective inhibitors against hCE1.

**Table 2:** The IC_50_ values of OA, UA, and their derivatives toward hCE1 and hCE2.

**Compound**	**IC_50_ (hCE2)^a^μM**	**IC_50_ (hCE1)^a^μM**	Selectivity^*^
**15**	16.09	0.13	123.07
**16**	6.12	2.41	2.54
**17**	5.70	6.23	0.91
**18**	4.11	3.21	1.28
**19**	10.17	0.19	53.53
**20**	56.04	0.017	3296.5
**21**	9.56	0.037	258.37
**22**	83.03	0.012	6919.2
**23**	11.93	1.83	6.52
**24**	>100	0.90	>111.11
**25**	11.92	33.21	0.36
**26**	5.64	8.94	0.63
**27**	3.16	6.94	0.45
**28**^b^	0.86	0.031	27.74

* *Selectivity is calculated from IC_50_ (hCE1)/IC_50_ (hCE2)*.

a *All data presented are averages of at least three separate experiments*.

b *Bis-p-nitrophenyl phosphate, a positive inhibitor against carboxylesterases*.

Consistently, 3-keto-UA derivative (**21**) exhibited similar trends in hCE1inhibition as 3-keto-OA derivative (**15**). Compound **22** (converting the C-3 hydroxyl group of UA to 3-O-β-carboxypropionyl group) showed excellent inhibitory effect against hCE1 with much lower IC_50_ value of 12 nM, which was 23-fold more potent than the parent compound UA and was 6,919-fold more selective over hCE2. Compound **23**, a UA derivative bearing an ester group at the C-28 site, showed reduced inhibitory effect and poor selectivity toward hCE1 in contrast to compound **2**. Compound **25**, an 11-keto-UA derivative, displayed moderate inhibitory effect on hCE2 but its inhibition against hCE1 was significantly reduced compared to compound **24**, suggesting that the carbonyl group introduced in such position was unbeneficial for hCE1 inhibition. Compounds **26** and **27**, converting the C-28 carboxyl group of compound **1** and **20** to C-30 carboxyl moiety, respectively, displayed enhanced inhibitory effects against hCE2, while their inhibitory effects toward hCE1 were dramatically decreased. These results suggested that C-30 carboxyl group was beneficial for hCE2 inhibition but not good for hCE1 inhibition. In addition, a known inhibitor bis-p-nitrophenyl phosphate (BNPP) was tested under identical conditions as a positive control (Umehara et al., 2016). The result indicated that BNPP showed inferior inhibitory effect and poor selectivity in contrast to compounds **20** and **22** (Table 2). To the best of our knowledge, compound **22** is the most potent and highly selective inhibitor against hCE1 reported to date.

**Figure 3:**
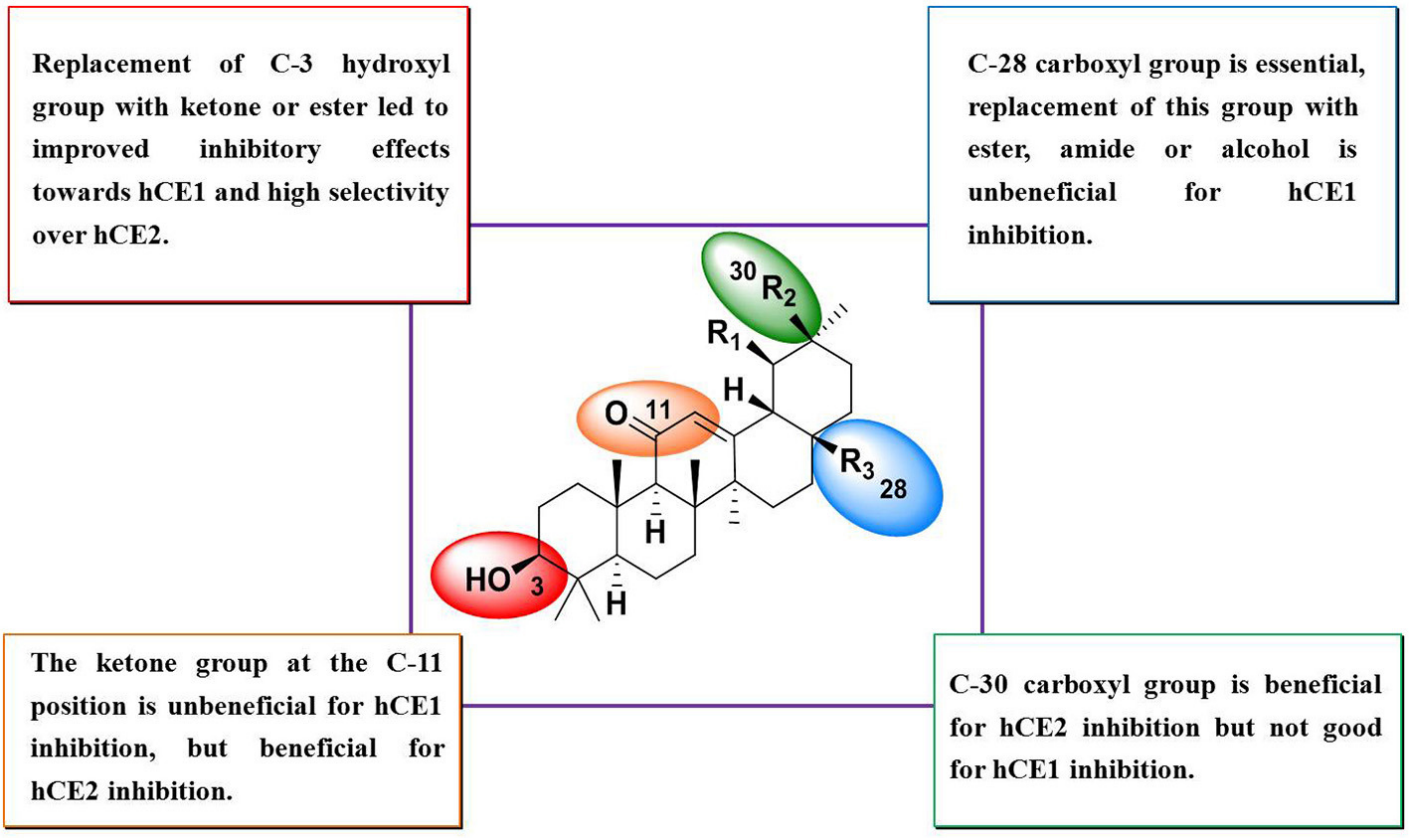
SAR summary of OA derivatives.

The structure-activity relationships (SAR) of these pentacyclic triterpenoids as hCE1 inhibitors have been summarized in Figure 3, which are very helpful for medical chemists to design and develop more potent and highly selective hCE1 inhibitors for biomedical applications.

### 3D-QSAR analysis

**Figure 4:**
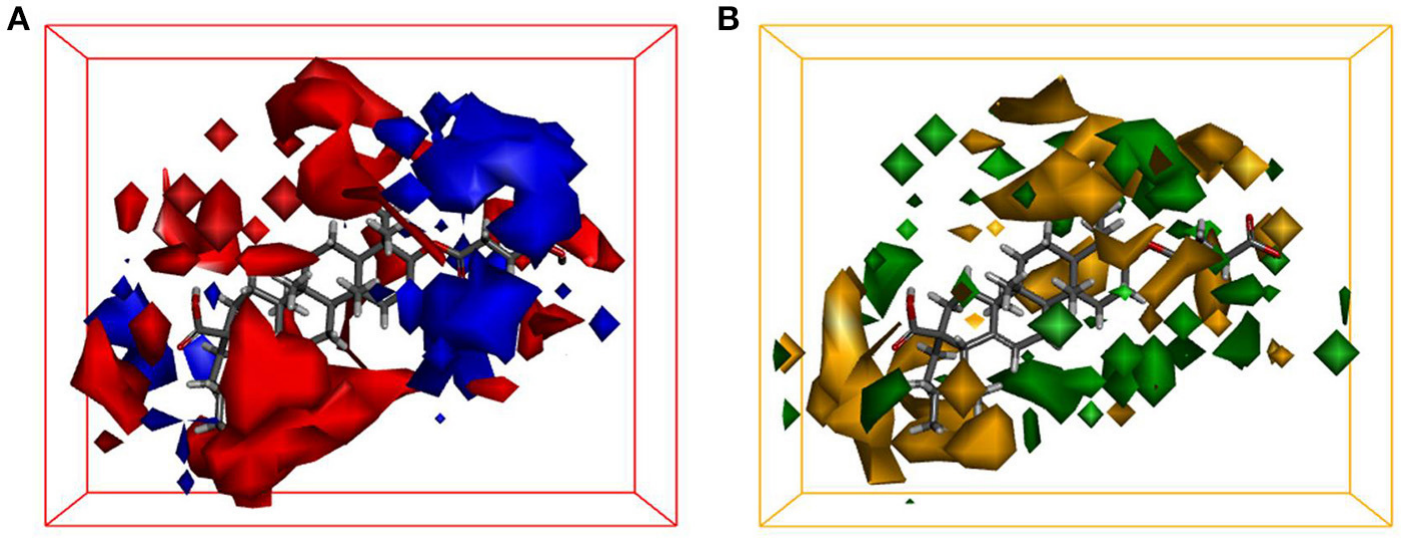
CoMFA steric and electrostatic contours displayed with most potent compound 22. **(A)** The red contours indicate the regions where substitution with more electronegative substituent are beneficial for hCE1 inhibition, whereas the blue contour shows the reverse; **(B)** The green areas indicate that the steric bulks are positively correlated with inhibitory activity, whereas the yellow are as indicate the steric bulks are negatively correlated with inhibitory activity.

**Figure 5:**
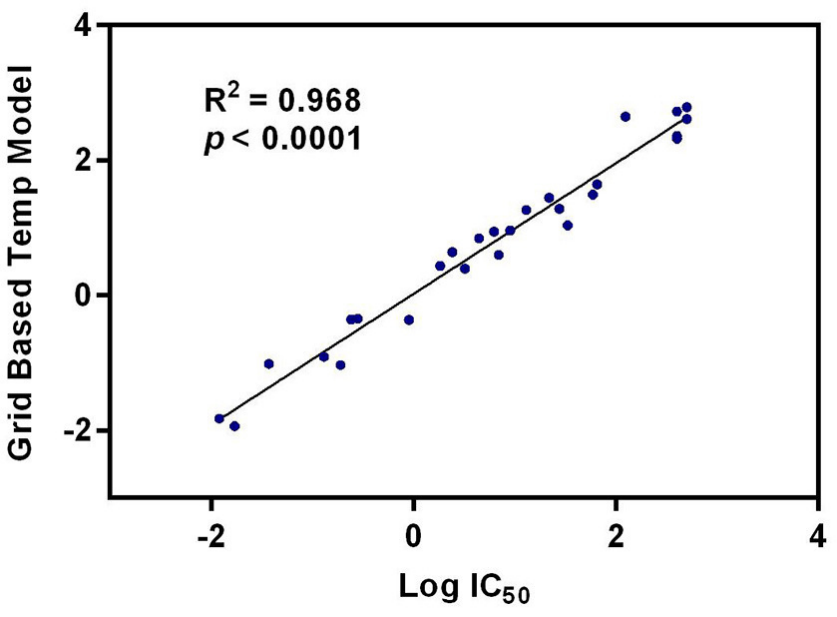
The correlation between the predictive and experimental inhibitory effects against hCE1 of compounds 1–27.

Quantitative Structure-Activity Relationship (QSAR) studies have been extensively applied to explore the correlations between biological activities and molecular descriptors for different classes of compounds (Soderholm et al., 2006; Vujasinovic et al., 2012). In this study, standard comparative molecular field analysis (CoMFA) was used to explore the relationships between structural features of all tested triterpenoids and their inhibitory effects on hCE1 (Table S1). The CoMFA steric and electrostatic fields based on PLS analysis were presented as 3-D contour plots in Figure 4. As shown in Figure 4A, the large red areas indicated that such regions with negative charges were favorable for hCE1 inhibition, while the blue are as implied that such regions with positive charges were favorable for hCE1 inhibition. The green-yellow steric contours depicted in Figure 4B illustrated that the steric bulks in green areas were beneficial for hCE1 inhibition, while the steric bulks in yellow areas were unbeneficial for hCE1 inhibition. The resulting CoMFA models suggested that both 3-D steric and electronic interactions could strongly affect the inhibitory effects of pentacyclic triterpenoids on hCE1. Notably, these findings agreed well with the experimental data, such as the electronegative group (red contour) at the C-28 position with the carboxyl group was beneficial for hCE1 inhibition, and the electropositive groups (blue contour) at the C-3 position could enhance the inhibitory effects against hCE1. The best CoMFA model was used to predict the inhibitory effects of all tested compounds in this study, which gave good statistical results (with the cross-validated *q*^2^ value of 0.554) and shown a strong correlation coefficient (*R*^2^ = 0.968) with experimental data (Figure 5). These findings provide new insights into the fine correlations between the inhibitory effects against hCE1 and the steric-electrostatic properties of triterpenoids, which are extremely helpful for rational design of novel pentacyclic triterpenoids as potent and selective hCE1 inhibitors.

### Inhibition kinetic analyses of compound 22 toward hCE1

**Figure 6:**
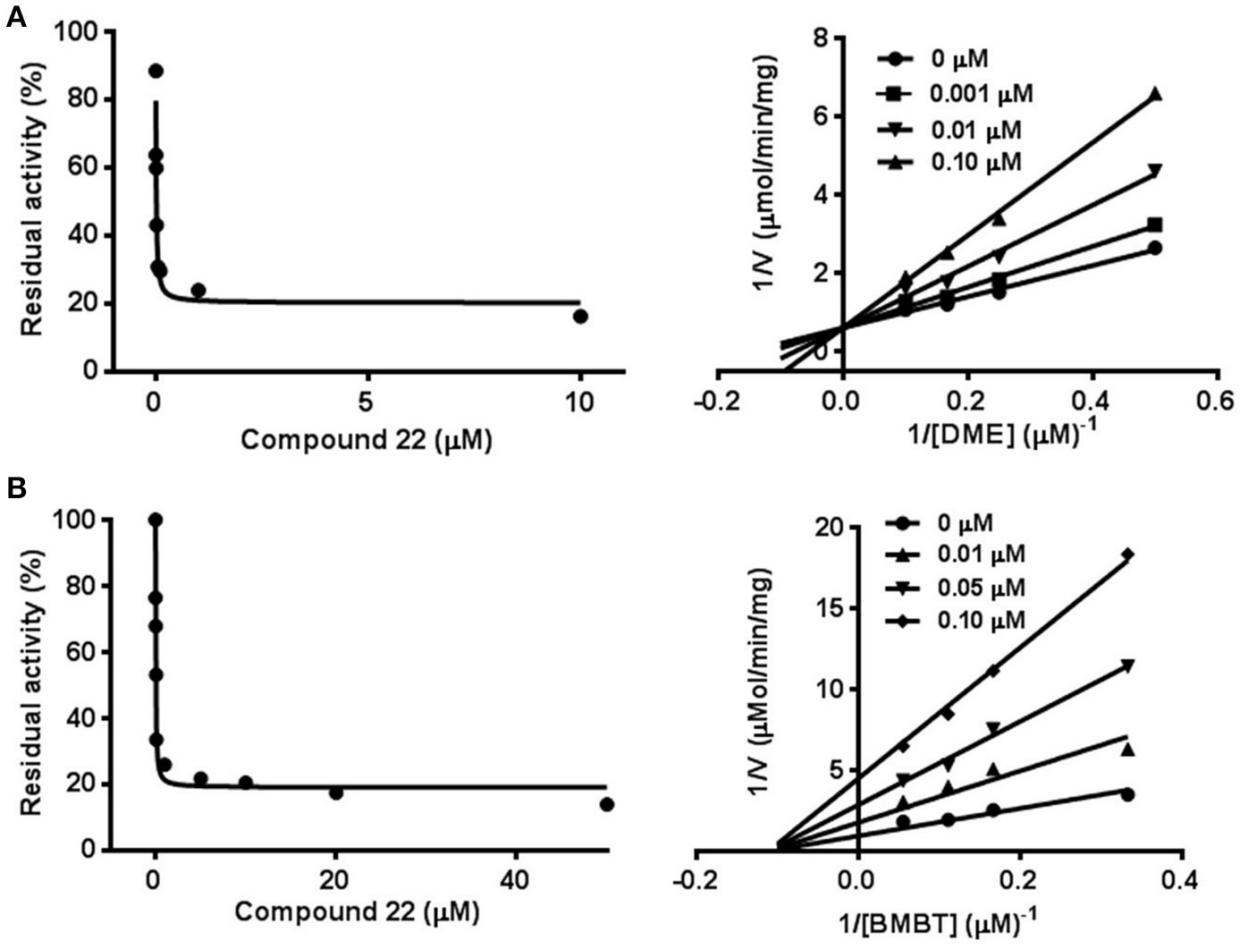
Inhibition behaviors of compound 22 against hCE1 mediated DME **(A)** and BMBT **(B)** hydrolysis. Left: the dose-dependent inhibition curves. Right: the Lineweaver-Burk plots. All data represent the mean of triplicate determinations.

To further investigate the inhibitory behaviors of compound **22** on the catalytic activity of hCE1, the inhibition behaviors of this compound against hCE1 were performed. As shown in Figure 6A, Lineweaver-Burk plots demonstrated that compound **22** could inhibit hCE1 in HLMs *via* competitive inhibition, with the *K*_i_ values of 12.6 nM. In addition, the inhibitory tendency and potency of compound **22** against DME-hydrolysis in both HLMs and hCE1 were much closed (Figure S1). The IC_50_ value of compound **22** against recombinant hCE1 was evaluated as 9.2 nM, which has the similar value of inhibitory effect against hCE1 in HLMs (12.6 nM). These results demonstrated that compound **22** is highly selective and potent hCE1 inhibitors, which might be used as promising tools for exploring the biological functions of hCE1 in complex biological systems.

Taking into account that hCE1 had at least two different ligand-binding sites, it is necessary to identify the ligand-binding sites of compound **22** and to investigate whether compound **22** displayed potent inhibitory effect against hCE1-meidated hydrolysis of other substrates (Lei et al., 2017). To this end, another optical probe substrate (BMBT) for hCE1 was used for further investigation on the inhibition behaviors of compound **22** (Liu et al., 2014). As shown in Figure 6B, Lineweaver-Burk plot demonstrated that compound **22** could inhibit hCE1-mediated BMBT hydrolysis in HLMs *via* non-competitive manner, with the IC_50_ and *K*_i_ values as 30.3 and 35.5 nM in HLMs, respectively. These findings clearly demonstrated that compound **22** could bind on the same ligand-binding site as **DME** rather than BMBT site, but this compound also displayed potent inhibitory effects against hCE1-meidated BMBT hydrolysis.

### Molecular docking simulations

**Figure 7:**
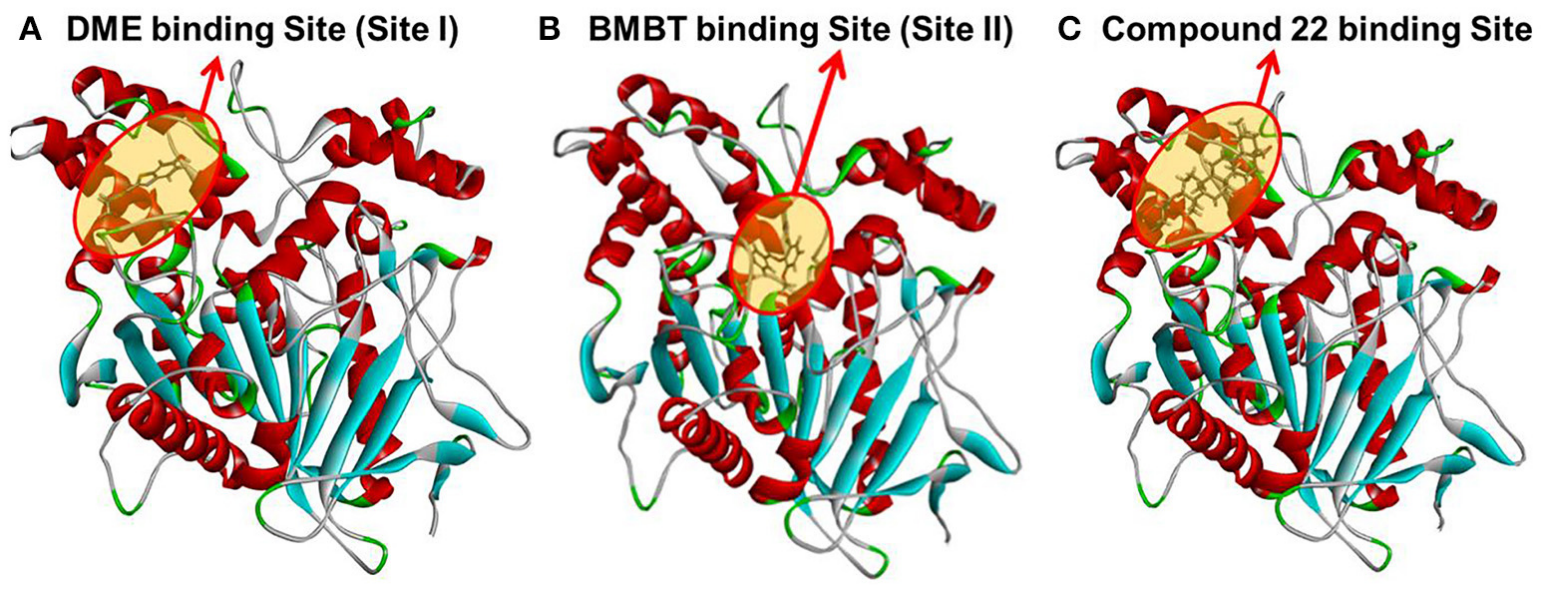
A stereo view of the crystal structure of hCE1 and the stereo diagram of each ligand aligned in its active site. **(A)** DME could bind on hCE1 at the ligand binding site I; **(B)** BMBT could bind on hCE1 at the ligand binding site II; **(C)** compound 22 could bind on hCE1 at the ligand binding site I (DME site).

In order to gain a deep understanding of the inhibitory behavior of compound **22** against hCE1 from the view of ligand-enzyme interactions, molecular docking simulations were performed using a previous reported crystal structure of hCE1 (PDB ID: 2DQY) as the macromolecular model. As shown in Figure 7, Figures S2, S3 (Supplementary Data), DME and compound **22** could bind on hCE1 at the same site (also called ligand-bind site I) which was a hydrophobic pocket located at the surface of the catalytic domain of hCE1 (Figures 7A,C), while the BMBT (also called ligand-bind site II) bound on hCE1 at another site which was surrounded by Ser221, Ile359, and His468, etc. These findings agreed well with the experimental results from inhibition kinetic analyses, in which compound **22** functioned as a competitive inhibitor against hCE1-mediated DME hydrolysis but was a non-competitive inhibitor against hCE1-mediated BMBT hydrolysis.

## Conclusions

In summary, a series of natural triterpenoids were collected and their inhibitory effects against human carboxylesterases were assayed. Two natural pentacyclic triterpenoids including OA and UA were found to display strong inhibitory effects on hCE1. Other natural pentacyclic triterpenoids, such as polygalacic acid (**3**) with more hydroxyl groups, glycyrrhetic acid (**4**), and celastrol (**7**) with carboxyl group at the C-30 site, displayed poor selectivity toward hCE1. Furthermore, in contrast to UA, β-boswellic acid (**5**) and 11-keto-β-boswellic acid (**6**) with carboxyl group at the C-23 site, demonstrated strong inhibitory effects on hCE2 and high selectivity over hCE1. Natural tetracyclic triterpenoids (**8–14**) exhibit less potentency and poor selectivity on hCE1, suggesting that the long alkyl chain at the C-20 site is unbeneficial for hCE1 inhibition. With the help of a series of semi-synthetic derivatives of OA and UA, the structure-activity relationships (SAR) analysis revealed that the carboxyl group at the C-28 site of OA and UA is very essential for hCE1 inhibition, and any modifications on this group with ester, amide or alcohol are unbeneficial for hCE1 inhibition. In contrast, the modifications of C-3 hydroxyl group are beneficial for hCE1 inhibition, and the replacement of C-3 hydroxyl group with a ketone or ester can lead to improvement of hCE1 inhibitory effects and high selectivity over hCE2. Guided by these SARs, the structural modifications of OA and UA, converting the C-3 hydroxyl group to 3-O-β-carboxypropionyl in compounds **20** and **22** lead to a dramatically increase of the inhibitory effects against hCE1 and high selectivity over hCE2. 3D-QSAR analysis demonstrated that electrostatic field and steric field are key factors affecting the inhibitory effects of OA and UA derivatives on hCE1. Further investigations demonstrated that compound **22** is a potent competitive inhibitor against hCE1-mediated DME hydrolysis but functioned as a noncompetitive inhibitor against BMBT hydrolysis. Molecular docking revealed that compound **22** could bind on the active site of hCE1 at the same ligand-binding site as DME, but this ligand-binding site is different from that of BMBT. All these findings will be very helpful for medicinal chemists to design and develop more potent and selective hCE1 inhibitors as drug candidates in future.

## Author contributions

LZ and GG were involved in the project design, carried out most of the experiments, and drafted the manuscript. TD contributed to the Molecular Docking simulations and 3D-QSAR analysis. PW, WL, ZW, JH, DW, and YF participated in the bioassays and inhibition kinetic analyses. WZ, GG, and LY designed and supervised this study. All authors read and approved the manuscript finally.

### Conflict of interest statement

The authors declare that the research was conducted in the absence of any commercial or financial relationships that could be construed as a potential conflict of interest.

## Abbreviations

BMBT: 2-(2-Hydroxy-3-methoxyphenyl) benzothiazole
BNPP: Bis-*p*-nitrophenyl phosphate
CEs: Mammalian carboxylesterases
CoMFA: Comparative molecular field analysis
DDAB: 6,8-dichloro-9,9-dimethyl-7-oxo-7,9-dihydroacridin-2-yl benzoate
DMAP: 4-Dimethylaminopyridine
DME: D-Luciferin methyl ester
hCE1: Human carboxylesterase 1
hCE2: Human carboxylesterase 2
HLM: Human liver microsomes
OA: Oleanolic acid
QSAR: Quantitative structure-activity relationship
SAR: Structure-activity relationships
TLC: Thin layer chromatography
UA: Ursolic acid.
