# Supplementary material for: Structure-Activity Relationships of Pentacyclic Triterpenoids as Potent and Selective Inhibitors against Human Carboxylesterase 1
Source: Front Pharmacol. 2017 Jun 30;8:435. doi: 10.3389/fphar.2017.00435 (PMC5491650; doi:10.3389/fphar.2017.00435)
Supplement: Supplementary file 1 [file Table1.DOCX]

**Table S1** 3D-QSAR statistical parameters

| **Compound** | **Clean energy^a^** | **Observed Log(IC_50__hCE1^b^)** | **Predicted Log(IC_50__hCE1 ^b^)** |
| --- | --- | --- | --- |
| **1** | 137.356 | -0.552842 | -0.342686 |
| **2** | 142.092 | -0.619789 | -0.353355 |
| **3** | 67.7132 | 1.81117 | 1.64776 |
| **4** | 104.995 | 1.1126 | 1.26471 |
| **5** | 199.88 | 2.60206 | 2.3243 |
| **6** | 204.046 | 2.09167 | 2.6474 |
| **7** | 153.07 | 0.646404 | 0.848341 |
| **8** | 63.5913 | 2.69897 | 2.61468 |
| **9** | 64.0934 | 2.69897 | 2.79235 |
| **10** | 126.834 | 1.33726 | 1.44765 |
| **11** | 122.632 | 1.44107 | 1.28687 |
| **12** | 136.957 | 1.7679 | 1.49729 |
| **13** | 142.061 | 2.60206 | 2.36138 |
| **14** | 106.581 | 2.60206 | 2.72408 |
| **15** | 114.841 | -0.886057 | -0.908017 |
| **16** | 84.6978 | 0.382017 | 0.644814 |
| **17** | 97.0297 | 0.794488 | 0.947412 |
| **18** | 137.398 | 0.506505 | 0.397015 |
| **19** | 98.1412 | -0.721246 | -1.03087 |
| **20** | 141.77 | -1.76955 | -1.93506 |
| **21** | 158.629 | -1.4318 | -1.01116 |
| **22** | 143.3 | -1.92082 | -1.82317 |
| **23** | 140.94 | 0.262451 | 0.437375 |
| **24** | 157.971 | -0.0457575 | -0.361988 |
| **25** | 162.017 | 1.52127 | 1.03756 |
| **26** | 96.9874 | 0.951338 | 0.964466 |
| **27** | 96.6641 | 0.841359 | 0.604629 |

^a^: kcal/mol

^b^: μM
