# Supplementary figures and images for: Structure-Activity Relationships of Pentacyclic Triterpenoids as Potent and Selective Inhibitors against Human Carboxylesterase 1
Source: Front Pharmacol. 2017 Jun 30;8:435. doi: 10.3389/fphar.2017.00435 (PMC5491650; doi:10.3389/fphar.2017.00435)

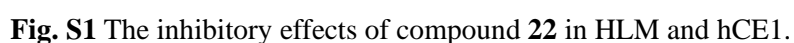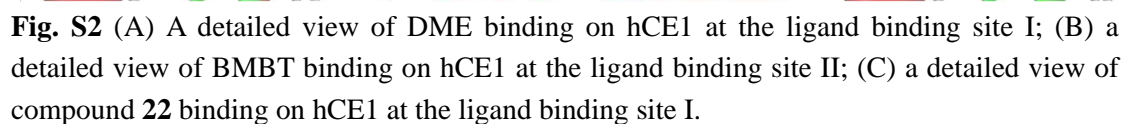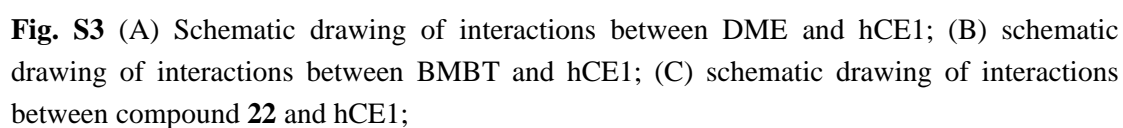

Supplement: Supplementary file 2 [file Presentation1.PDF]
