# Supplementary material for: Structure-Activity Relationships of Pentacyclic Triterpenoids as Potent and Selective Inhibitors against Human Carboxylesterase 1
Source: Front Pharmacol. 2017 Jun 30;8:435. doi: 10.3389/fphar.2017.00435 (PMC5491650; doi:10.3389/fphar.2017.00435)
Supplement: Supplementary file 3 [file Presentation2.PDF]

NMR spectra for compounds

3-Oxo-olean-12-en-28-oic acid (**15**): ^{1}H NMR (400 MHz, $CDCl_3$) δ 5.30 (s, 1H), 2.84 (dd, J = 13.7, 4.1 Hz, 1H), 2.59-2.50 (m, 1H), 2.42-2.31 (m, 1H), 2.04-1.82 (m, 5H), 1.77-1.55 (m, 7H), 1.54-1.17 (m, 12H), 1.15 (s, 3H), 1.08 (s, 3H), 1.05 (s, 3H), 1.03 (s, 3H), 0.93 (s, 3H), 0.91 (s, 3H), 0.87-0.83 (m, 3H), 0.81 (s, 3H).^{13}C NMR (101 MHz, $CDCl_3$) δ 217.7, 183.6, 143.7, 122.4, 55.3, 47.4, 46.9, 46.6, 45.8, 41.7, 41.1, 39.3, 39.1, 36.8, 34.1, 33.8, 33.1, 32.4, 32.2, 30.7, 27.7, 26.5, 25.8, 23.6, 23.8, 22.9, 21.4, 19.6, 17.0, 15.0.

3β-Olean-12-ene-3,28-diol (**16**): ^{1}H NMR (400 MHz, DMSO) δ 5.11 (s, 1H), 4.30 (s, 2H), 3.39 (s, 1H), 3.31 (d, J = 10.5 Hz, 1H), 3.02-2.98 (m, 1H), 2.94 (d, J = 10.5 Hz, 1H), 1.93 (dd, J = 13.7, 3.9 Hz, 1H), 1.85-1.61 (m, 5H), 1.54 -1.33 (m, 8H), 1.32-1.18 (m, 4H), 1.09 (d, J = 18.5 Hz, 5H), 0.97 (d, J = 16.5 Hz, 1H), 0.90 (s, 3H), 0.89 (s, 3H), 0.87 (s, 3H), 0.85 (s, 3H), 0.83 (s, 3H), 0.69-6.61 (m, 4H).^{13}C NMR (101 MHz, DMSO) δ 144.8, 121.9, 77.3, 67.9, 55.2, 47.5, 46.8, 42.3, 41.7, 38.9, 37.0, 36.9, 34.3, 33.5, 32.6, 31.4, 31.2, 28. 7, 26.1, 25.6, 24.0, 23.5, 18.5, 16.9, 16.5, 15.8.

3β-Hydroxy-olean-12-en-28-oic acidmethyl ester (**17**): ^{1}H NMR (400 MHz, $CDCl_3$) δ 5.28 (s, 1H), 3.62 (s, 3H), 3.21 (dd, J = 11.1, 4.4 Hz, 1H), 2.86 (dd, J = 13.7, 3.9 Hz, 1H), 2.03-1.82 (m, 3H), 1.73-1.50 (m, 12H), 1.46-1.37 (m, 2H), 1.35-1.25 (m, 4H), 1.20-1.16 (m, 2H), 1.13 (s, 3H), 1.07-1.04(m, 1H), 0.99 (s, 3H), 0.93 (s, 3H), 0.91 (s, 3H), 0.90 (s, 3H), 0.78 (s, 3H), 0.72 (s, 3H).^{13}C NMR (101 MHz, $CDCl_3$) δ 178.3, 143.8, 122.4, 79.0, 55.3, 51.5, 47.7, 46.7, 45.9, 41.7, 41.3, 39.3, 38.8, 38.5, 37.1, 33.9, 33.1, 32.7, 32.4, 30.7, 28.1, 27.7, 27.2, 25.9, 23.6, 23.4, 23.1, 18.3, 16.8, 15.6, 15.3.

3β-Hydroxy-olean-12-en-28-amide (**18**): ^{1}H NMR (400 MHz, DMSO) δ 6.76 (d, J = 5.2 Hz, 2H), 5.21 (s, 1H), 4.27 (s, 1H), 3.05-2.91 (m, 1H), 2.73 (dd, J = 13.2, 3.7 Hz, 1H), 1.92-1.84 (m, 1H), 1.82-1.79 (m, 2H), 1.67-1.61 (m, 2H), 1.55-1.37(m, 9H), 1.37-1.18 (m, 4H), 1.09 (s, 5H), 0.89 (s, 5H), 0.88 (s, 3H), 0.86 (s, 3H), 0.85 (s, 3H), 0.72 (s, 3H), 0.68 (s, 3H).13C NMR (101 MHz, DMSO) δ 179.27, 144.69, 121.76, 77.30, 55.30, 47.59, 46.62, 45.66, 41.75, 40.98, 39.33, 38.85, 38.54, 37.07, 34.15, 33.42, 33.09, 32.93, 30.90, 28.71, 27.43, 26.11, 23.99, 23.38, 22.86, 18.50, 17.40, 16.48, 15.57.

3β-O-Acetyl-olean-12-en-28-oic acid (**19**): ^{1}H NMR (400 MHz, DMSO) δ 5.16 (s, 1H), 4.45-4.34 (m, 1H), 2.76-2.73 (m, 1H), 2.00 (s, 3H), 1.97-1.87 (m, 1H), 1.91-1.80 m, 2H), 1.68-1.41 (m,

10H), 1.33-1.16 (m, 6H), 1.11 (s, 3H), 1.07-0.98 (m, 4H), 0.89 (s, 3H), 0.87 (s, 6H), 0.82 (s, 6H), 0.72 (s, 3H).

3β-O-(β-Carboxypropionyl)-olean-12-en-28-oic acid (**20**): ^{1}H NMR (400 MHz, DMSO) δ 12.04 (brs, 2H), 5.13 (s, 1H), 4.41 (dd, J = 11.4, 4.7 Hz, 1H), 2.51-2.47 (m, 4H), 2.11 (d, J = 11.3 Hz, 1H), 1.98-1.76 (m, 4H), 1.63-1.39 (m, 11H), 1.34-1.26 (m, 4H), 1.06 (s, 3H), 1.03-0.95 (m, 2H), 0.91 (s, 6H), 0.87-0.83 (m, 1H), 0.81 (s, 9H), 0.76 (s, 3H).^{13}C NMR (101 MHz, DMSO) δ 179.0, 173.9, 172.1, 144.3, 121.9, 80.5, 55.0, 47.3, 46.1, 45.9, 41.8, 41.3, 38.0, 37.8, 37.0, 33.3, 32.6, 30.9, 29.7, 29.2, 28.2, 27.7, 26.0, 23.8, 23.6, 23.4, 23.1, 18.3, 17.3, 17.1, 15.5.

3-Oxo-urs-12-en-28-oic acid (**21**): ^{1}H NMR (400 MHz, $CDCl_3$) δ 5.26 (s, 1H), 2.64-2.46 (m, 1H), 2.39-2.35 (m, 1H), 2.20 (d, J = 11.2 Hz, 1H), 2.07-1.82 (m, 5H), 1.75-1.58 (m, 4H), 1.58-1.19 (m, 11H), 1.13 (s, 1H), 1.09 (s, 6H), 1.06 (s, 3H), 1.03 (s, 3H), 0.96 (s, 3H), 0.86 (d, J = 6.4 Hz, 3H), 0.83 (s, 3H).^{13}C NMR (101 MHz, CDCl3) δ 217.8, 183.4, 138.1, 125.6, 55.3, 52.6, 48.0, 47.4, 46.8, 42.1, 39.5, 39.3, 39.1, 38.8, 36.7, 36.7, 34.2, 32.5, 30.6, 28.0, 26.6, 24.1, 23.5, 23.4, 21.5, 21.2, 19.6, 17.0, 17.0, 15.2.

3β-O-(β-Carboxypropionyl)-urs-12-en-28-oic acid (**22**): ^{1}H NMR (400 MHz, DMSO) δ 12.04 (brs, 2H), 5.13 (s, 1H), 4.41 (dd, J = 11.4, 4.7 Hz, 1H), 2.50- 2.45 (m, 4H), 2.11 (d, J = 11.3 Hz, 1H), 1.99-1.75 (m, 4H), 1.64-1.40 (m, 11H), 1.34-1.26 (m, 4H), 1.06 (s, 3H), 1.02-0.98 (m, 2H), 0.91 (s, 6H), 0.87-0.85 (m, 1H), 0.81 (s, 9H), 0.76 (s, 3H).^{13}C NMR (101 MHz, DMSO) δ 178.8, 173.9, 172.1, 138.7, 124.9, 80.5, 55.0, 52.8, 47.3, 42.5, 42.1, 39.0, 38.9, 38.1, 37.8, 36.9, 36.8, 33.0, 30.6, 29.7, 29.3, 28.2, 28.0, 24.3, 23.7, 23.3, 21.5, 18.2, 17.5, 17.4, 17.1, 15.6, 12.5, 11.9.

3β-Hydroxy-urs-12-en-28-oic acid methyl ester (**23**): ^{1}H NMR (400 MHz, $CDCl_3$) δ 5.25 (s, 1H), 3.60 (s, 3H), 3.33-3.12 (m, 1H), 2.23 (d, J = 11.4 Hz, 1H), 2.04-1.96 (m, 1H), 1.92-1.89 (m, 2H), 1.82-1.73 (m, 1H), 1.69-1.57 (m, 7H), 1.55-1.45 (m, 5H), 1.40-1.21 (m, 7H), 1.08 (s, 3H), 0.99 (s, 3H), 0.95-0.93 (m, 6H), 0.87-0.84 (m, 5H), 0.78 (s, 3H), 0.75 (s, 3H).^{13}C NMR (101 MHz, $CDCl_3$) δ 178.1, 138.2, 125.6, 79.1, 55.2, 52.9, 51. 5, 48.1, 47.6, 42.0, 39.5, 39.1, 38.9, 38.8, 38.6, 37.0, 36.7, 33.0, 30.7, 29.7, 28.1, 28.0, 27.3, 24.2, 23.6, 23.3, 21.2, 18.3, 17.0, 16.9, 15.6, 15.4.

3-Oxo-urs-12-en-28-oic acid methyl ester (**24**): ^{1}H NMR (400 MHz, DMSO) δ 5.17 (s, 1H), 3.52 (s, 3H), 2.34-2.27 (m, 1H), 2.19-2.15 (m, 1H), 2.06-1.95 (m, 1H), 1.92-1.88 (m, 2H), 1.85-1.64 (m, 2H), 1.64-1.15 (m, 16H), 1.06 (s, 3H), 1.00 (s, 3H), 0.98 (s, 3H), 0.95 (s, 3H), 0.92 (s, 3H), 0.82 (d, J = 6.5 Hz, 3H), 0.72 (s, 3H).

3,11-Dioxo-urs-12-en-28-oic acid methyl ester (**25**): ^{1}H NMR (400 MHz, $CDCl_3$) δ 5.64 (s, 1H), 3.62 (s, 3H), 3.09-2.88 (m, 1H), 2.67-2.59 (m, 1H), 2.54-2.26 (m, 3H), 2.13-2.06 (m, 1H), 1.90-1.73 (m, 3H), 1.70 -1.35 (m, 9H), 1.31 (s, 3H), 1.26 (s, 6H), 1.09 (s, 3H), 1.06 (s, 3H), 0.98 (s, 3H), 0.95 (s, 3H), 0.87 (d, 3H).^{13}C NMR (101 MHz, CDCl3) δ 217.1, 199.1, 177.2, 163.3, 130.6, 60.7, 55.4, 52.8, 51.9, 47.7, 47.7, 44.5, 43.9, 39.8, 38.6, 36.8, 36.0, 34.2, 32.4, 30.3, 28.5, 26.5, 23.9, 21.4, 21.1, 21.0, 18.8, 17.1, 15.5.

3β-O-(β-Carboxypropionyl)-olean-12-en-30-oic acid (**27**): ^{1}H NMR (400 MHz, DMSO) δ 12.13 (s, 2H), 5.17 (s, 1H), 4.42 (dd, J = 11.4, 4.6 Hz, 1H), 2.50-2.45 (m, 4H), 2.06-1.94 (m, 1H), 1.92-1.82 (m, 3H), 1.78-1.66 (m, 3H), 1.63-1.49 (m 7H), 1.44-1.23 (m, 5H), 1.13 (s, 3H), 1.07 (s, 3H), 1.03-0.96 (m, 2H), 0.92 (s, 6H), 0.87-0.85 (m, 1H), 0.82 (s, 6H), 0.74 (s, 3H).13C NMR (101 MHz, DMSO) δ 178.5, 174.6, 172.1, 144.9, 122.2, 80.4, 54.9, 48.3, 47.3, 43.6, 42.9, 41.6, 38.5, 38.1, 37.8, 36.8, 32.5, 32.1, 31.1, 29.6, 28.7, 28.6, 28.1, 26.8, 26.2, 26.1, 23.6, 23.6, 18.2, 17.1, 17.0, 15.7.

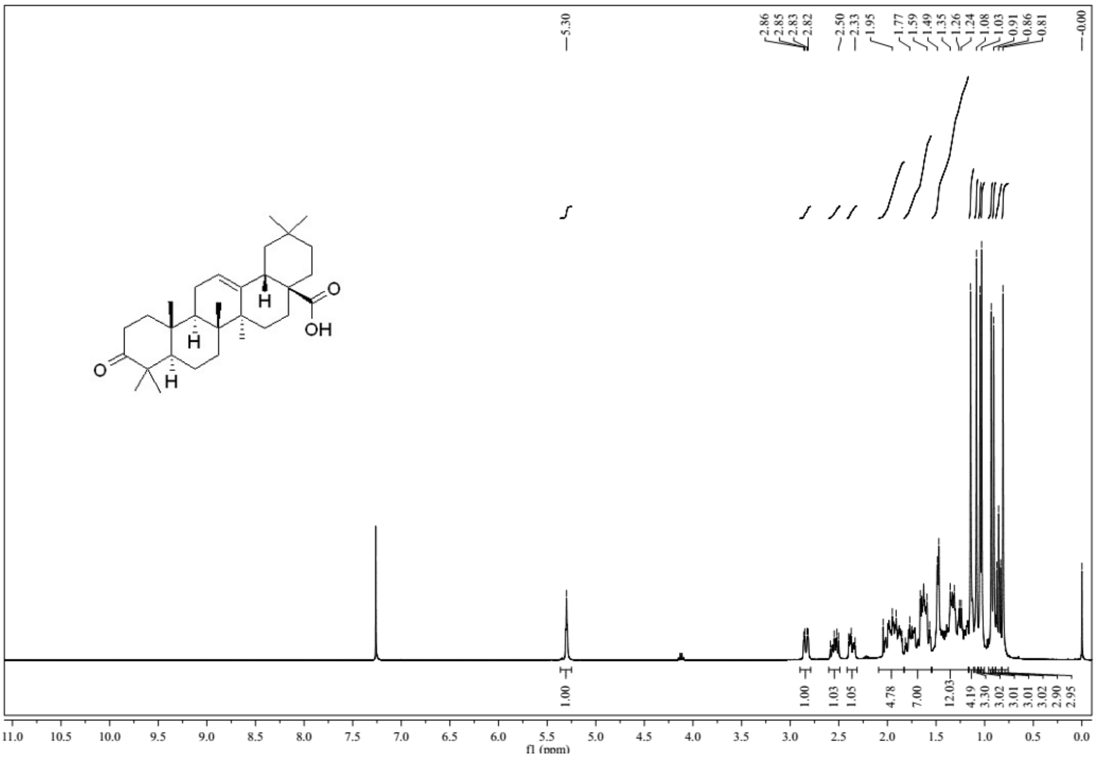

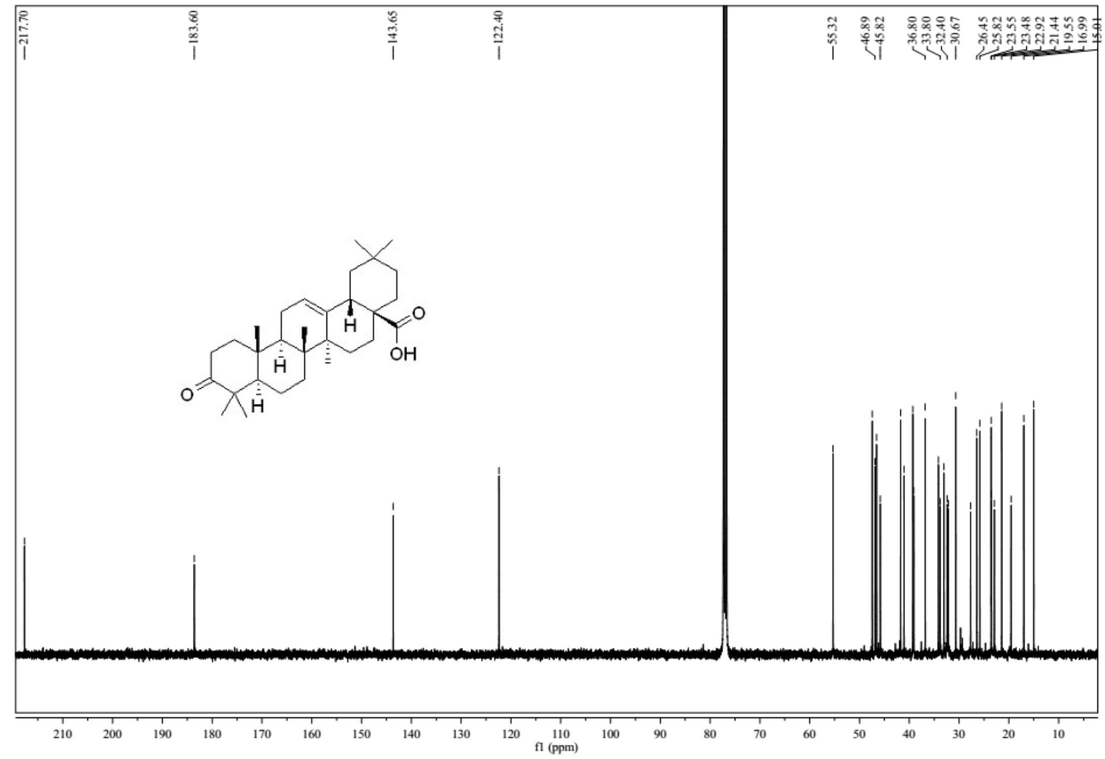
217.70
183.60
143.65
122.40
55.32
46.89
45.82
36.80
33.80
32.40
30.67
26.45
25.82
23.55
23.48
22.92
21.44
19.55
16.99
15.01
OH
O
H
f1 (ppm)

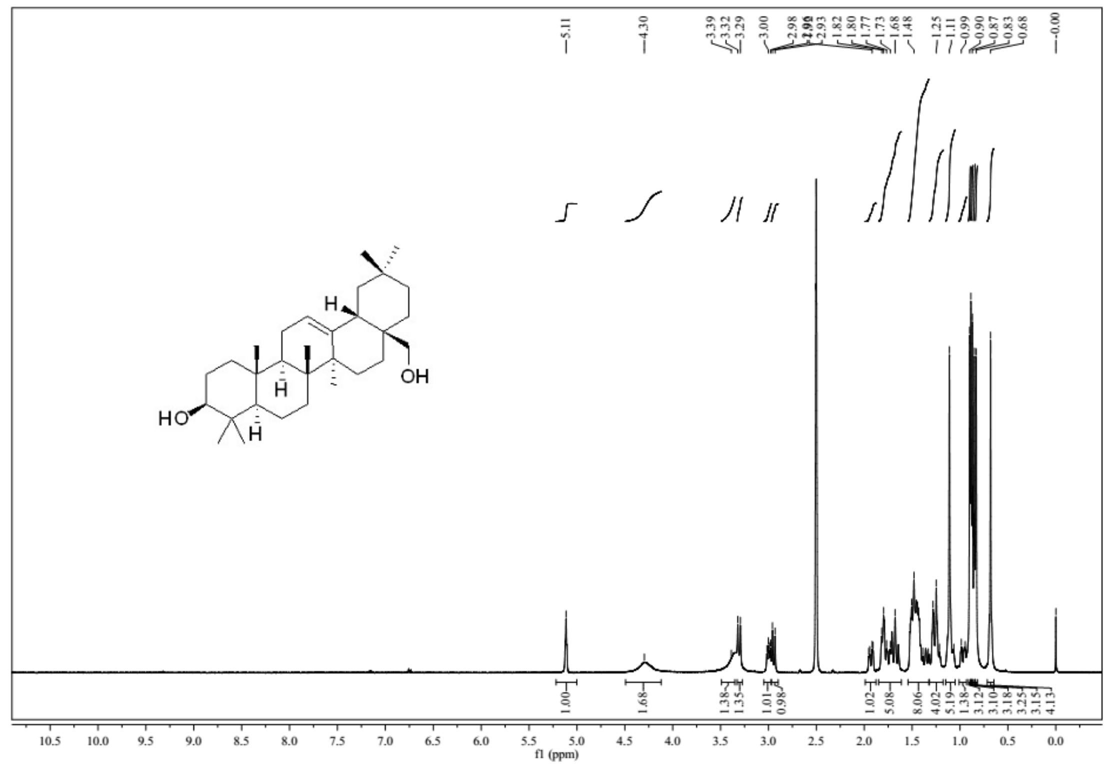
5.11
4.30
3.39
3.32
3.29
3.00
2.98
2.93
1.82
1.80
1.77
1.73
1.68
1.48
1.25
1.11
0.99
0.90
0.87
0.83
0.68
-0.00
HO
OH
H
f1 (ppm)

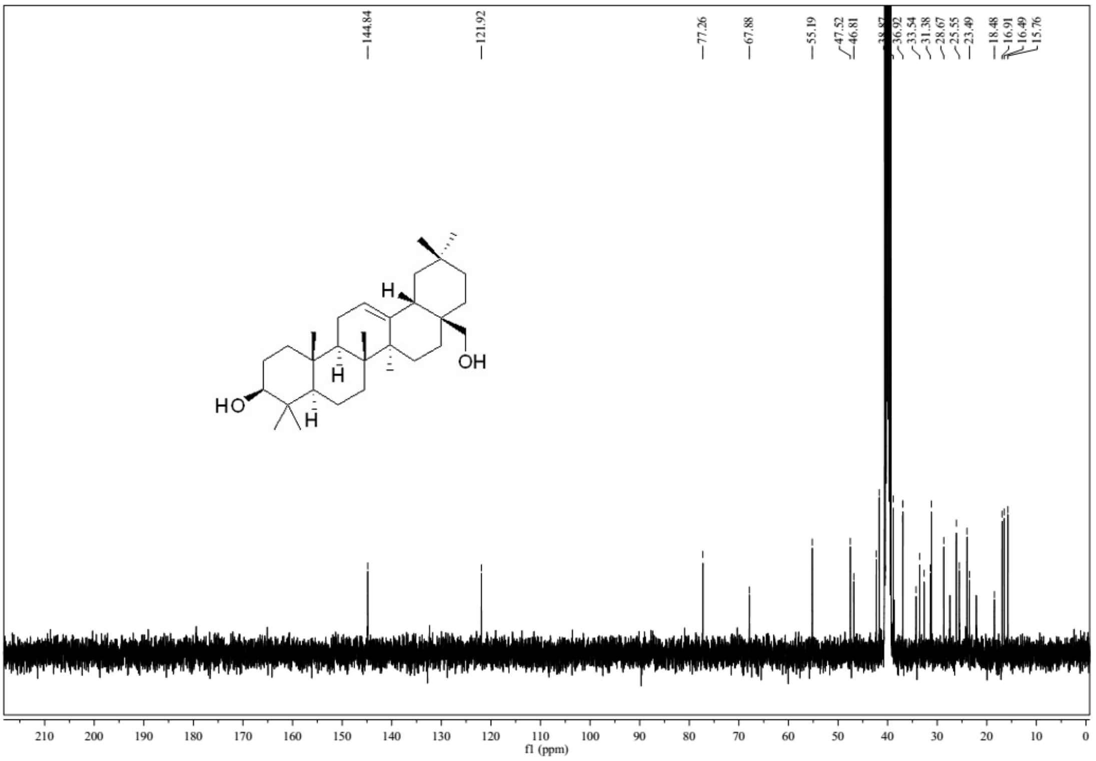
144.84
121.92
77.26
67.88
55.19
47.52
46.81
36.92
33.54
31.38
28.67
25.55
23.49
18.48
16.91
16.49
15.76
H
HO
OH
f1 (ppm)

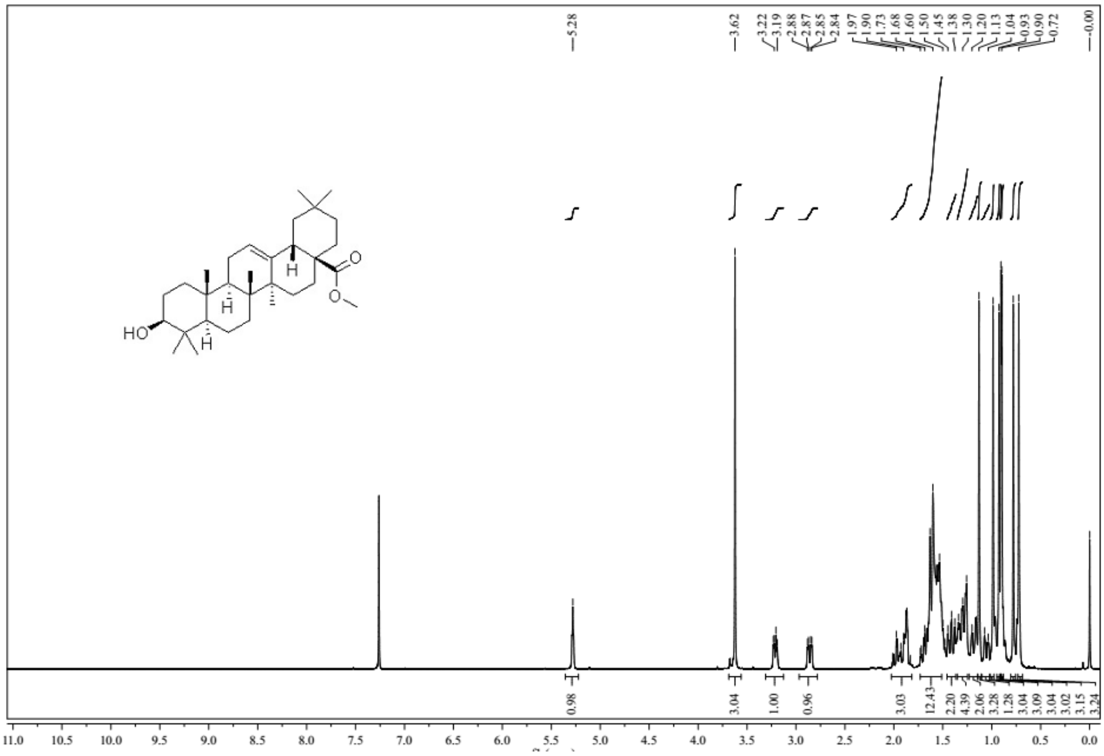
5.28
3.62
3.22
3.19
2.88
2.87
2.85
2.84
1.97
1.90
1.73
1.68
1.60
1.50
1.45
1.38
1.30
1.20
1.13
1.04
0.93
0.90
0.72
-0.00
HO
H
O
0.98
3.04
1.00
0.96
3.03
12.43
2.20
4.39
2.06
3.28
1.28
3.04
3.09
3.04
3.02
3.15
3.24

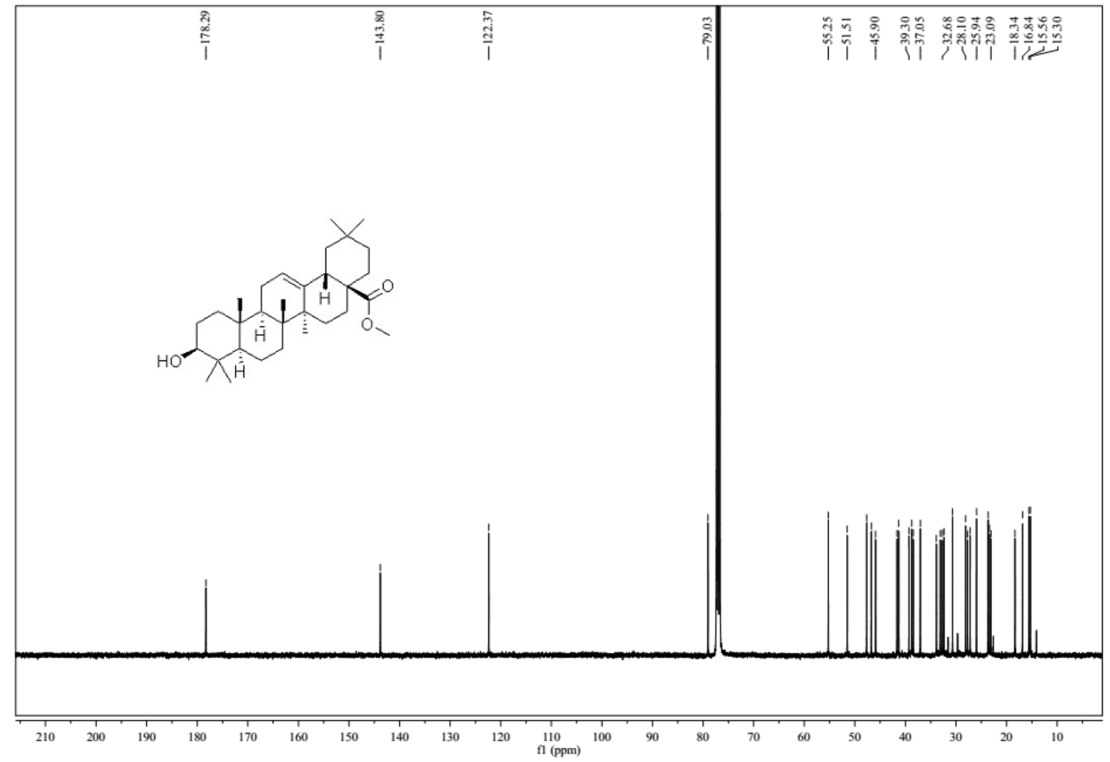
178.29
143.80
122.37
79.03
55.25
51.51
45.90
39.30
37.05
32.68
28.10
25.94
23.09
18.34
16.84
15.56
15.30
HO
H
O
f1 (ppm)

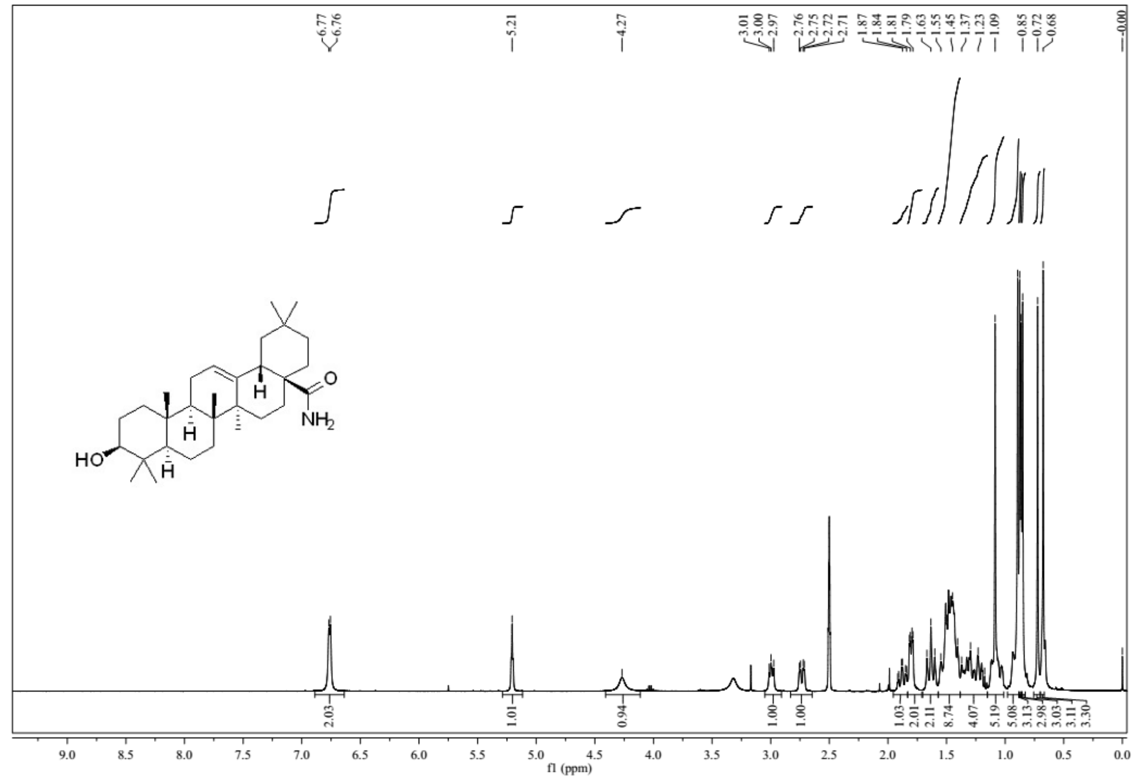
6.77
6.76
5.21
4.27
3.01
3.00
2.97
2.76
2.75
2.72
2.71
1.87
1.84
1.81
1.79
1.63
1.55
1.45
1.37
1.23
1.09
0.85
0.72
0.68
0.00
HO
H
O
NH2
2.03
1.01
0.94
1.00
1.00
1.03
2.01
2.11
8.74
4.07
5.19
5.08
3.13
2.98
3.03
3.11
3.30
f1 (ppm)

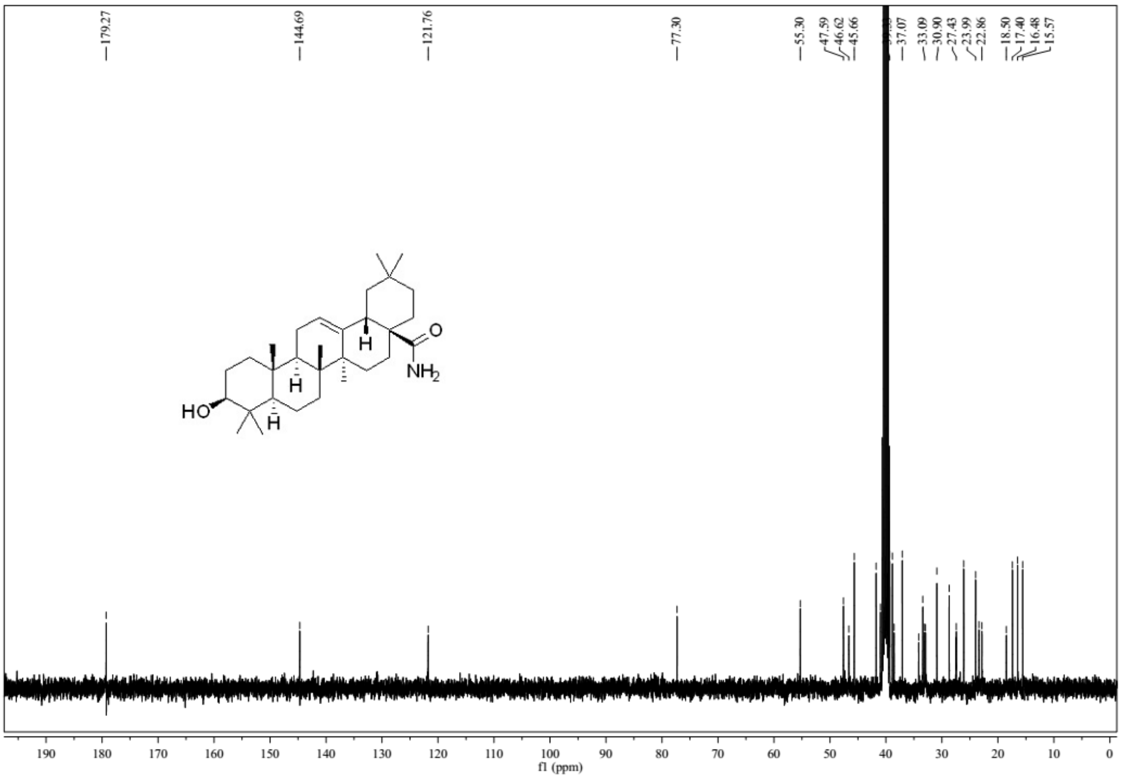
179.27
144.69
121.76
77.30
55.30
47.59
46.62
45.66
37.07
33.09
30.90
27.43
23.99
22.86
18.50
17.40
16.48
15.57
HO
H
H
O
NH2
190 180 170 160 150 140 130 120 110 100 90 80 70 60 50 40 30 20 10 0
f1 (ppm)

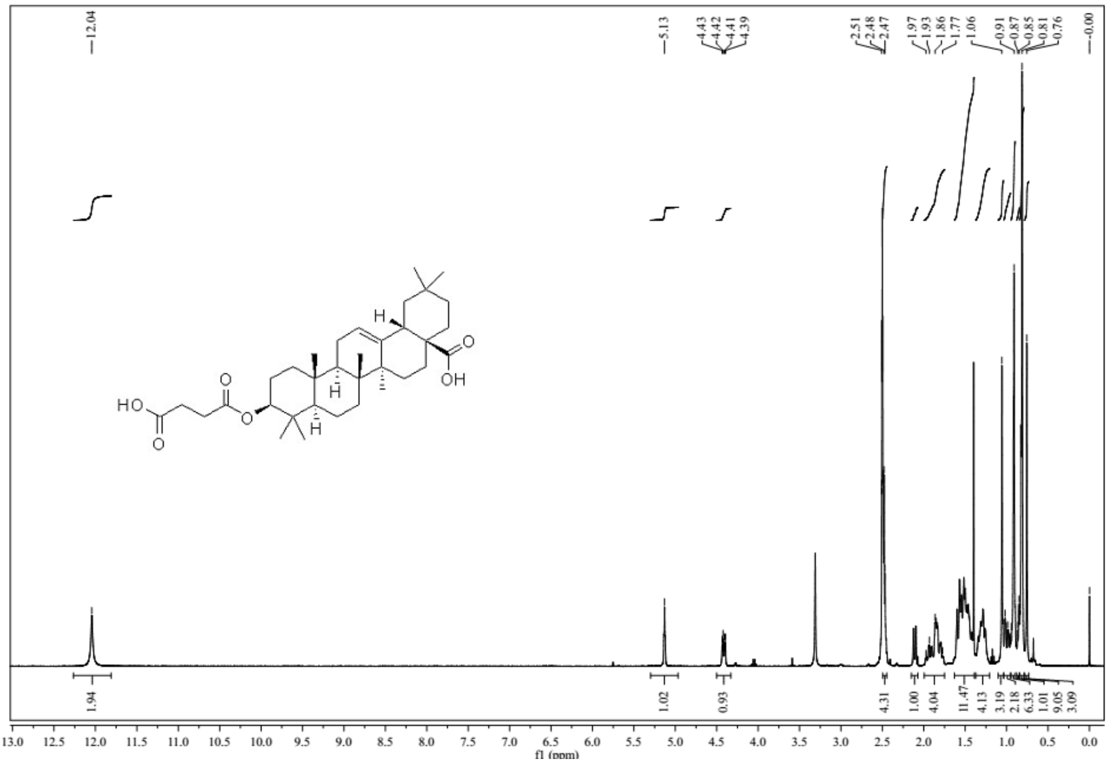
12.04
5.13
4.43
4.42
4.41
4.39
2.51
2.48
2.47
1.97
1.93
1.86
1.77
1.06
0.91
0.87
0.85
0.81
0.76
-0.00
HO
O
O
O
H
H
OH
1.94
1.02
0.93
4.31
1.00
4.04
11.47
4.13
3.19
2.18
6.33
1.01
9.05
3.09
13.0 12.5 12.0 11.5 11.0 10.5 10.0 9.5 9.0 8.5 8.0 7.5 7.0 6.5 6.0 5.5 5.0 4.5 4.0 3.5 3.0 2.5 2.0 1.5 1.0 0.5 0.0
f1 (ppm)

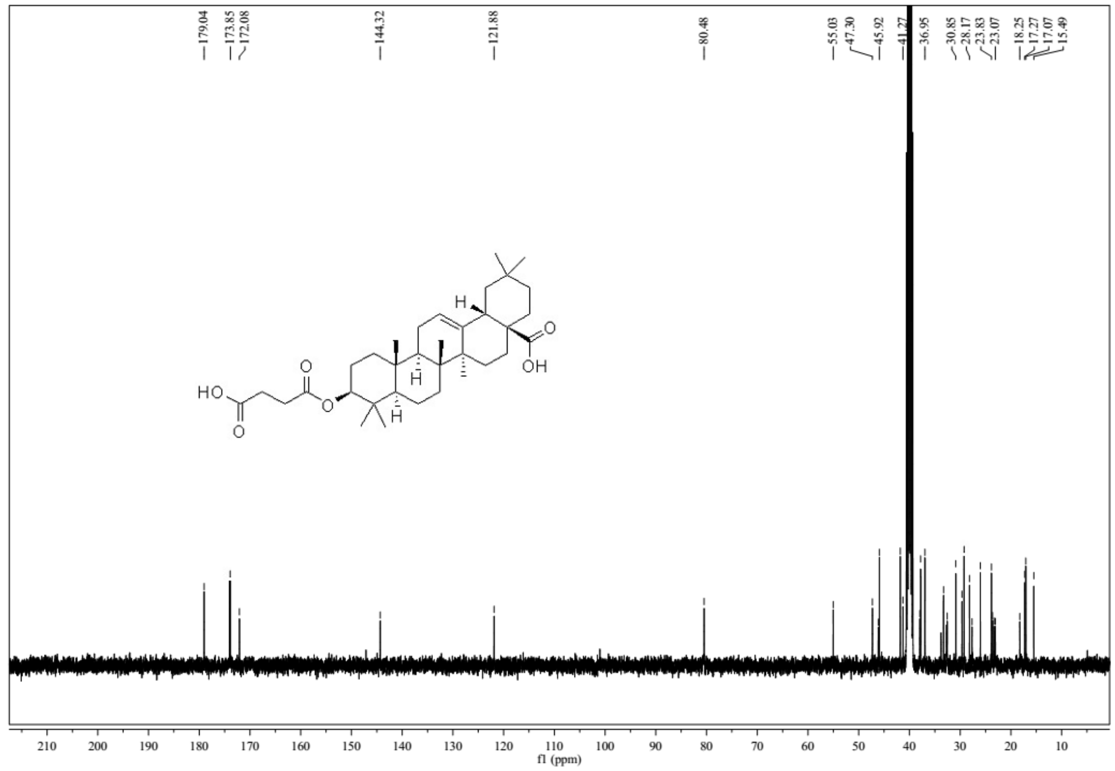
179.04
173.85
172.08
144.32
121.88
80.48
55.03
47.30
45.92
41.27
36.95
30.85
28.17
23.83
23.07
18.25
17.27
17.07
15.49
210
200
190
180
170
160
150
140
130
120
110
100
90
80
70
60
50
40
30
20
10
f1 (ppm)
HO
O
O
O
H
H
H
O
OH

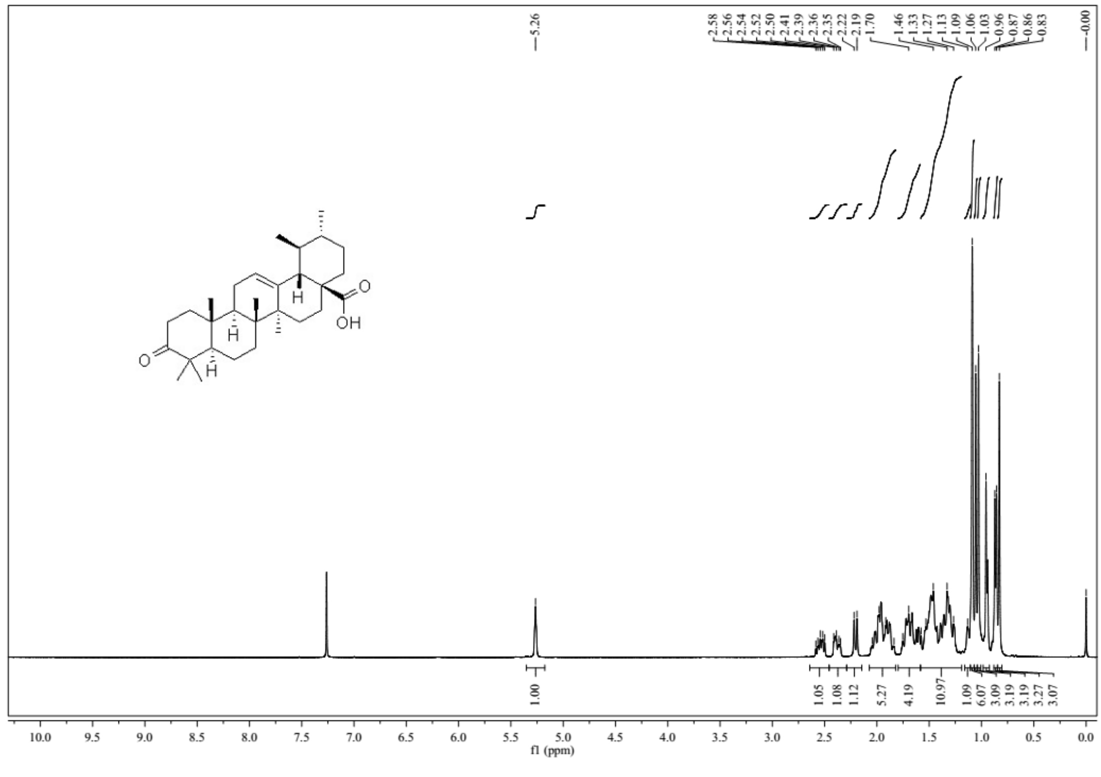
5.26
2.58
2.56
2.54
2.52
2.50
2.41
2.39
2.36
2.35
2.22
2.19
1.70
1.46
1.33
1.27
1.13
1.09
1.06
1.03
0.96
0.87
0.86
0.83
-0.00
1.00
1.05
1.08
1.12
5.27
4.19
10.97
1.09
6.07
3.09
3.19
3.27
3.07
10.0
9.5
9.0
8.5
8.0
7.5
7.0
6.5
6.0
5.5
5.0
4.5
4.0
3.5
3.0
2.5
2.0
1.5
1.0
0.5
0.0
f1 (ppm)
O
H
H
H
O
OH

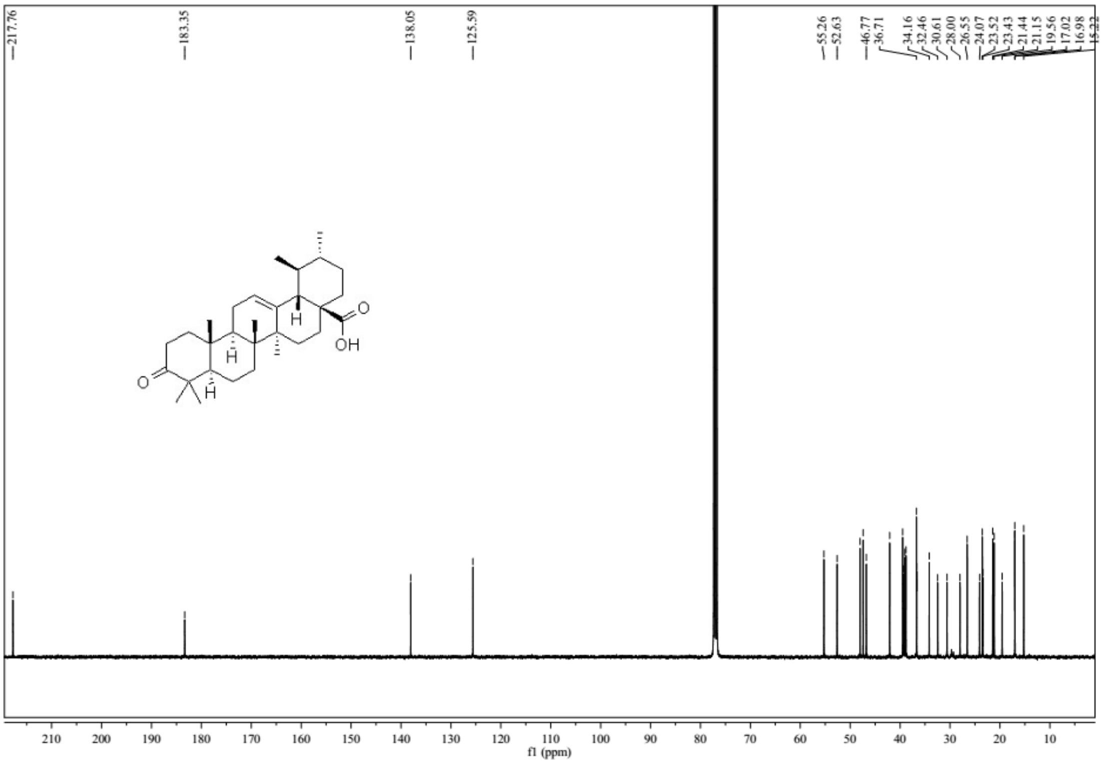

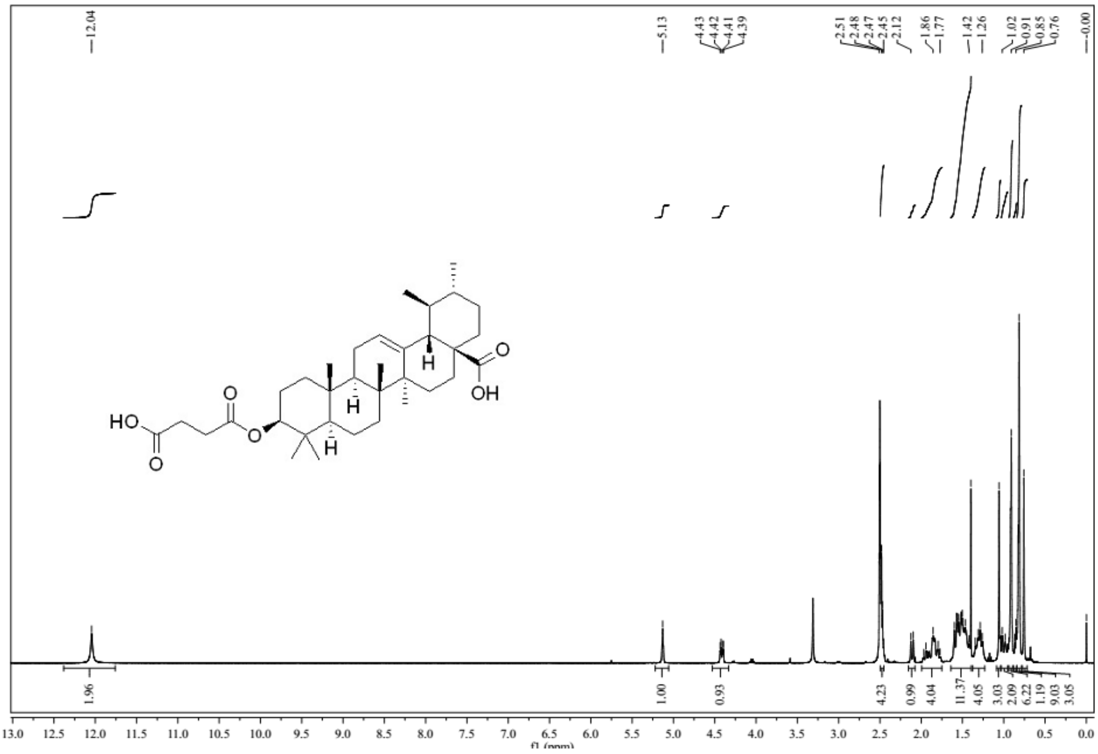

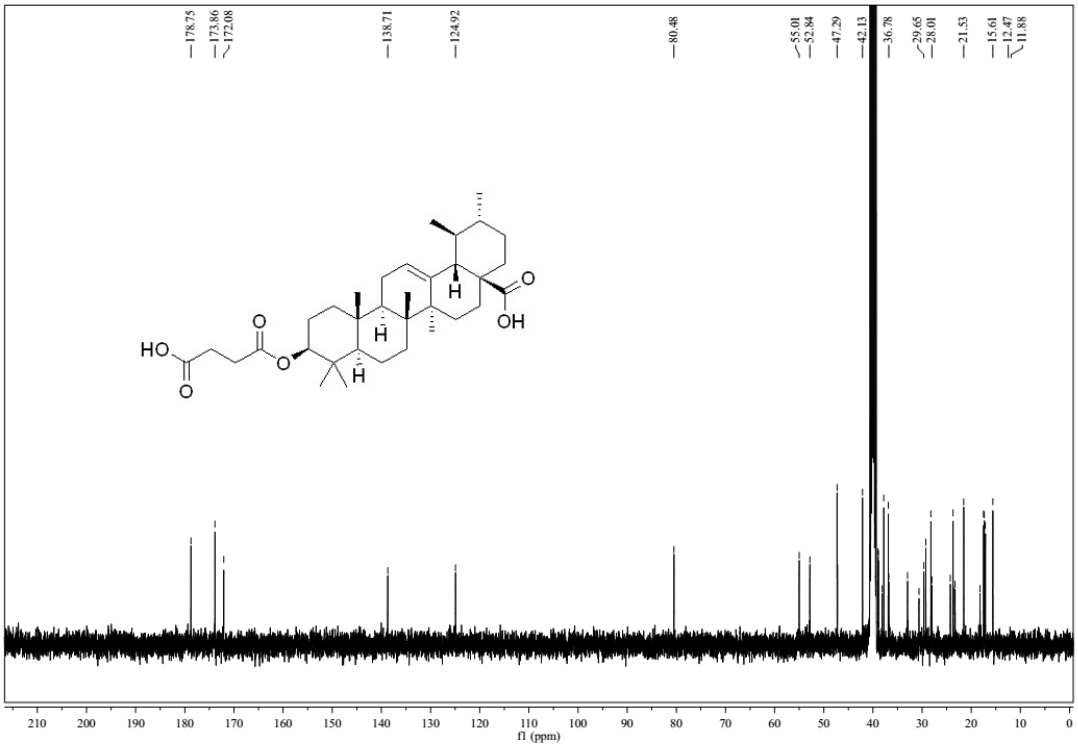
178.75
173.86
172.08
138.71
124.92
80.48
55.01
52.84
47.29
42.13
36.78
29.65
28.01
21.53
15.61
12.47
11.88
HO
O
O
O
H
H
H
O
OH
f1 (ppm)

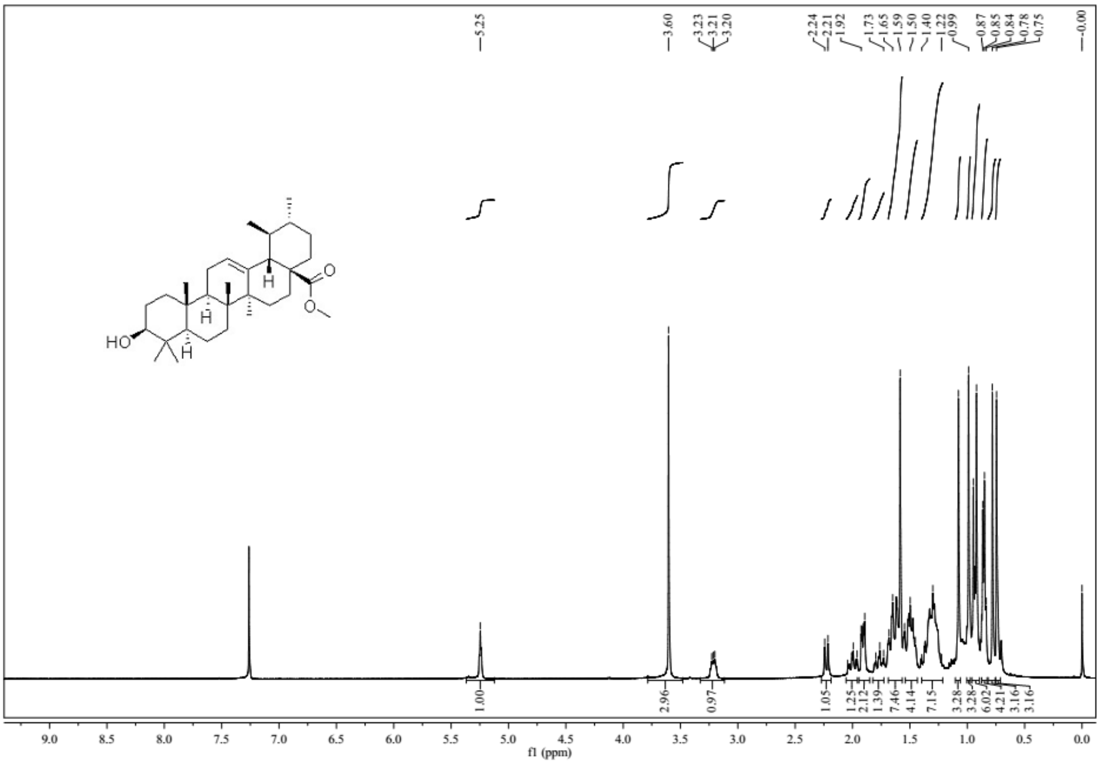
5.25
3.60
3.23
3.21
3.20
2.24
2.21
1.92
1.73
1.65
1.59
1.50
1.40
1.22
0.99
0.87
0.85
0.84
0.78
0.75
-0.00
HO
H
H
H
O
O
1.00
2.96
0.97
1.05
1.25
2.12
1.39
7.46
4.14
7.15
3.28
3.28
6.02
4.21
3.16
3.16
f1 (ppm)

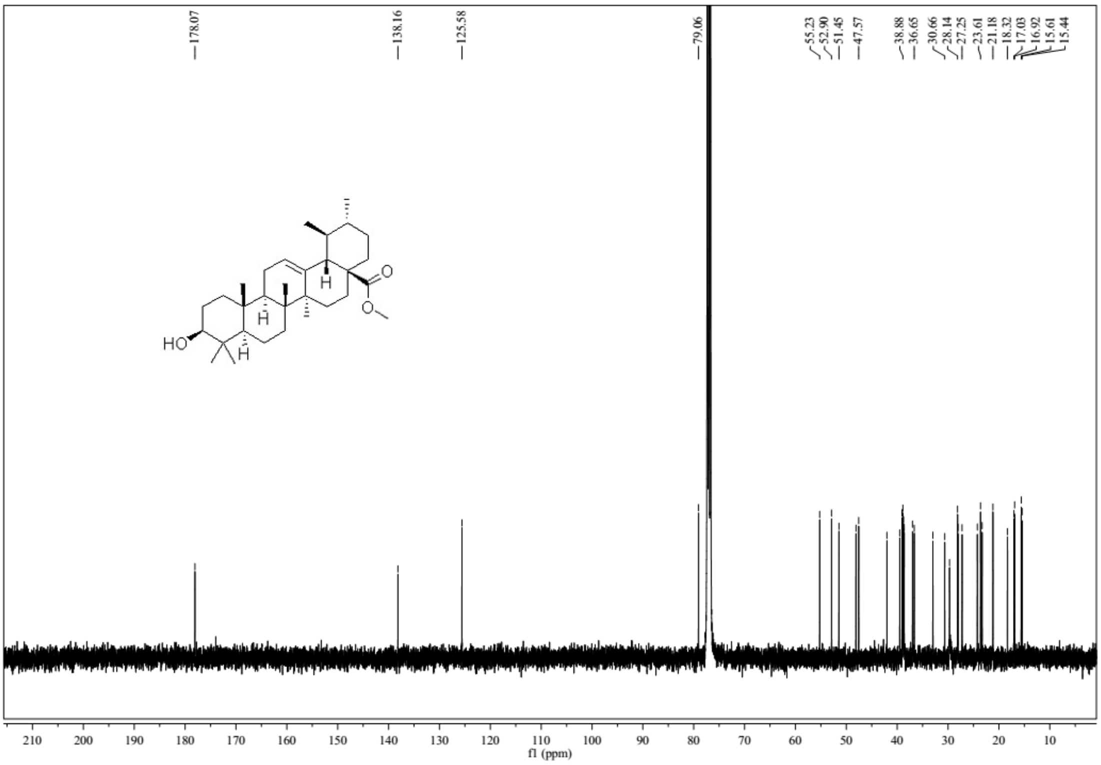
178.07
138.16
125.58
79.06
55.23
52.90
51.45
47.57
38.88
36.65
30.66
28.14
27.25
23.61
21.18
18.32
17.03
16.92
15.61
15.44
HO
H
H
H
O
O
f1 (ppm)

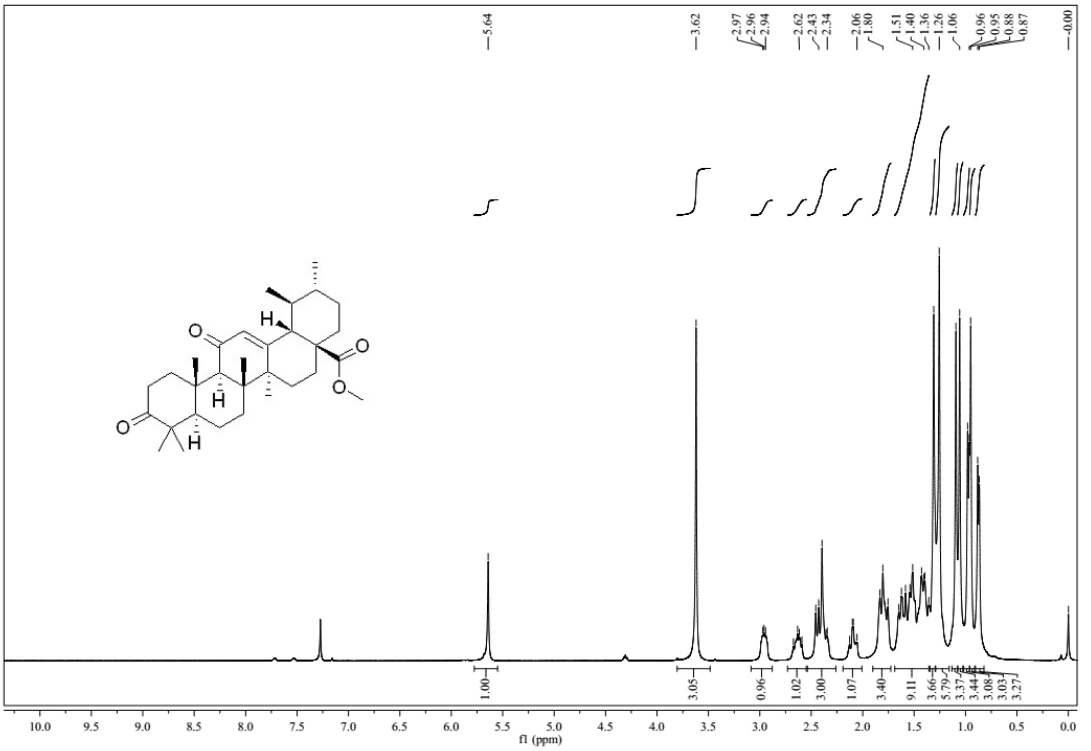
5.64
3.62
2.97
2.96
2.94
2.62
2.43
2.34
2.06
1.80
1.51
1.40
1.36
1.26
1.06
0.96
0.95
0.88
0.87
0.00
1.00
3.05
0.96
1.02
3.00
1.07
3.40
9.11
3.66
5.79
3.37
3.44
3.08
3.03
3.27
O
O
O
O
H
H
H
f1 (ppm)

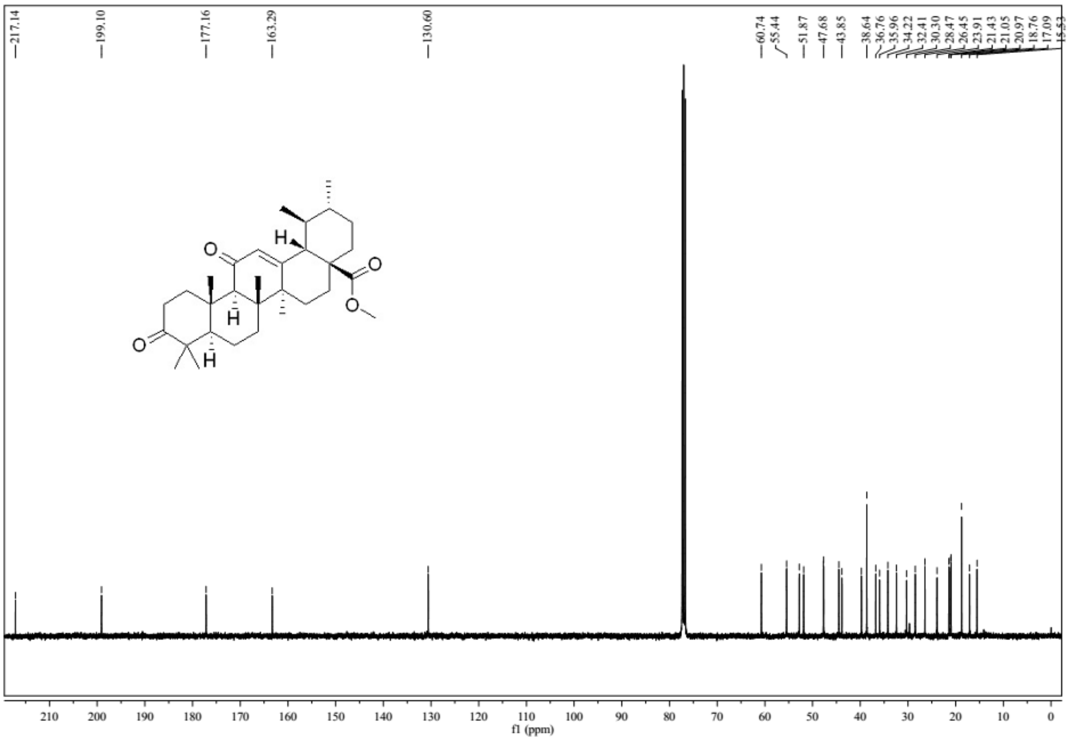

217.14
199.10
177.16
163.29
130.60
60.74
55.44
51.87
47.68
43.85
38.64
36.76
35.96
34.22
32.41
30.30
28.47
26.45
23.91
21.43
21.05
20.97
18.76
17.09
210 200 190 180 170 160 150 140 130 120 110 100 90 80 70 60 50 40 30 20 10 0
f1 (ppm)

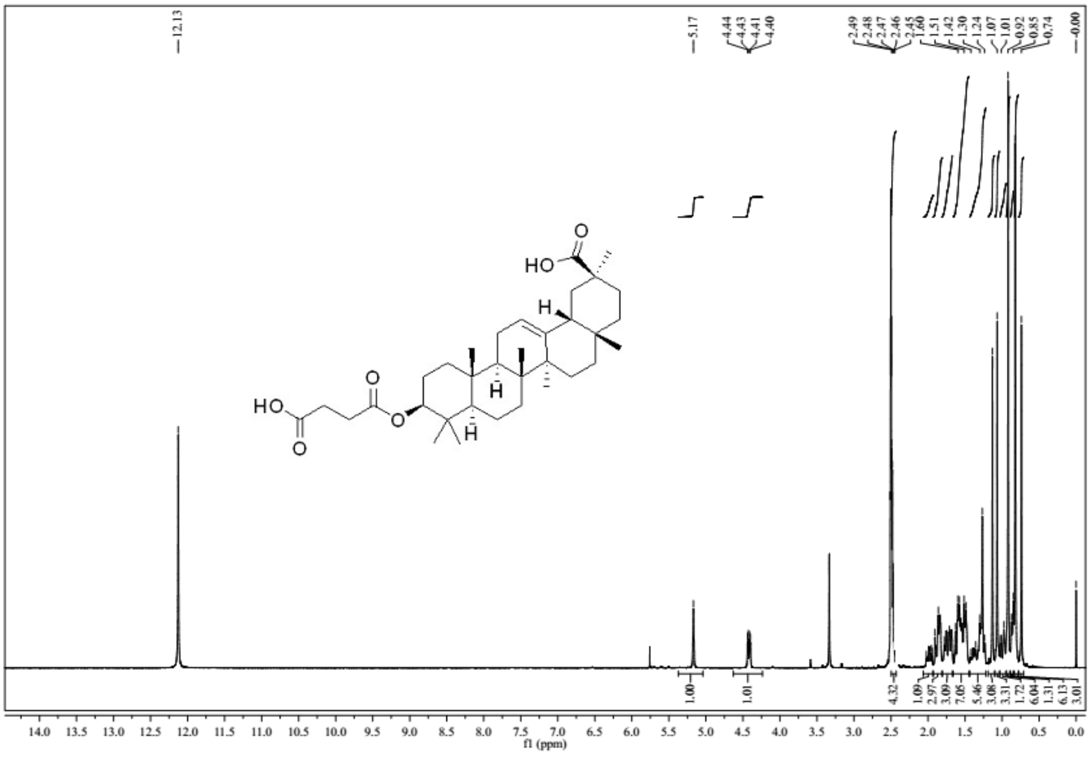

12.13
5.17
4.44
4.43
4.41
4.40
2.49
2.48
2.47
2.46
2.45
1.60
1.51
1.42
1.30
1.24
1.07
1.01
0.92
0.85
0.74
0.00
HO
O
H
1.00
1.01
4.32
14.0 13.5 13.0 12.5 12.0 11.5 11.0 10.5 10.0 9.5 9.0 8.5 8.0 7.5 7.0 6.5 6.0 5.5 5.0 4.5 4.0 3.5 3.0 2.5 2.0 1.5 1.0 0.5 0.0
f1 (ppm)

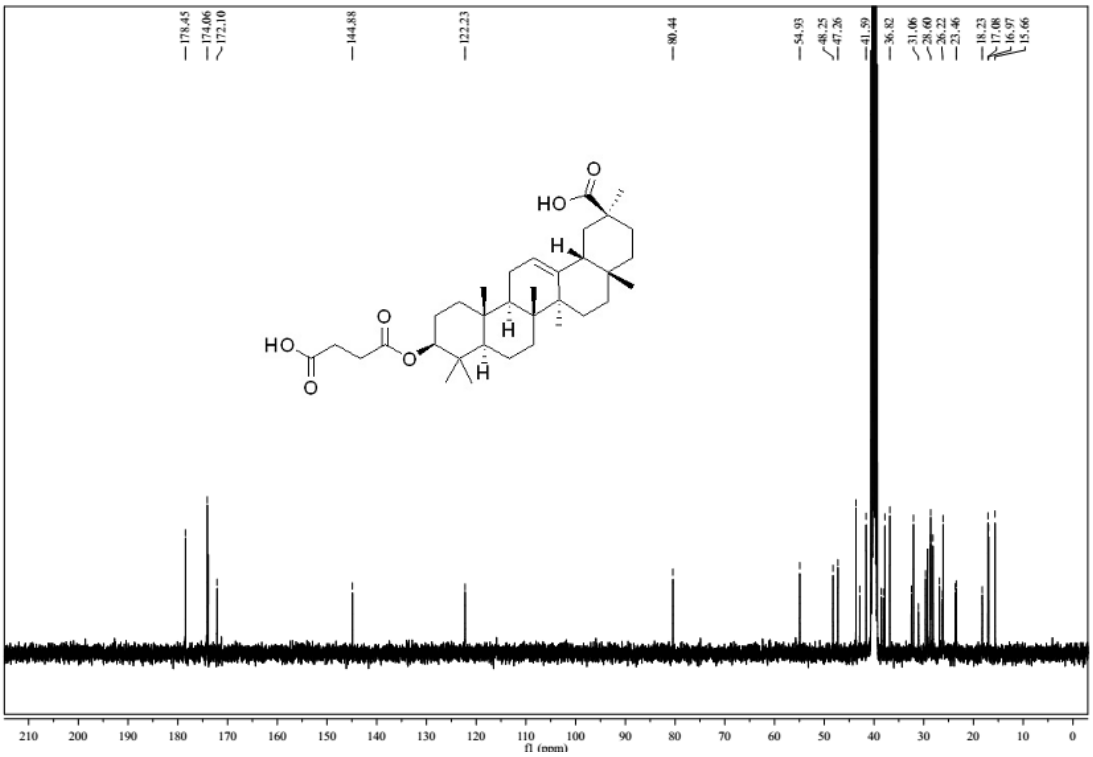
178.45
174.06
172.10
144.88
122.23
80.44
54.93
48.25
47.26
41.59
36.82
31.06
28.60
26.22
23.46
18.23
17.08
16.97
15.66
HO
O
H
210 200 190 180 170 160 150 140 130 120 110 100 90 80 70 60 50 40 30 20 10 0
f1 (ppm)
